# Confined Mg^2+^-Directed Interfacial Evolution of Primary Structural Units of Mg-MOF-74 on Platelet Clinoptilolite for CO_2_/CH_4_, CH_4_/N_2_, and CO_2_/N_2_ Separations

**DOI:** 10.3390/gels12100902

**Published:** 2026-10-07

**Authors:** Nan Sun, Tiejun Ma, Shengquan Liu, Tallat Munir, Bang Xu, Jihong Sun

**Affiliations:** Beijing Key Laboratory for Green Catalysis and Separation, Beijing University of Technology, Beijing 100124, China; sunnan@emails.bjut.edu.cn (N.S.); matiejun@bjut.edu.cn (T.M.); liushengqaun@emails.bjut.edu.cn (S.L.); ch023tm@kust.edu.pk (T.M.)

**Keywords:** sol–gel species, Mg^2+^-exchanged clinoptilolite, confined metal source, interfacial growth, gas adsorption separation

## Abstract

Controlling MOF nucleation on zeolite surfaces remains challenging when metal ions are freely supplied in solution, often resulting in poorly regulated interfaces. Herein, Mg^2+^-exchanged platelet clinoptilolite (Mg-PCP) was used as a confined metal source to direct the in situ growth of Mg-MOF-74-related sol–gel species (primary structural units, abbreviated as PSUs) without adding external Mg^2+^ salts. Using 2,5-dihydroxyterephthalic acid (DHTA) as a ligand, the structure features of the prepared PSU@Mg-PCPs revealed the progressive evolution of the disordered Mg-DHTA coordination species into locally ordered, unsaturated Mg-MOF-74-related interfacial subunits, accompanied by gradual interfacial roughening and coordination ordering. The evolving interfaces regulated the adsorption and separation behaviors of CO_2_, CH_4_, and N_2_. At 298 K, PSU@Mg-PCP-10 exhibited apparent CO_2_/CH_4_ and CO_2_/N_2_ separation factors of 24.23 and 15.01, respectively, higher than those of Mg-PCP (7.83 and 9.01). Meanwhile, PSU@Mg-PCP-180 achieved the highest CO_2_ uptake of 1.60 mmol g^−1^ at 273 K. Grand canonical Monte Carlo simulations indicated that the separation behaviors originated from the combined effects of micropore blockage and preferential CO_2_ adsorption at Mg^2+^ sites and newly formed interfacial regions. This confined-metal-source strategy provides a metal-efficient route for tailoring zeolite–MOF interfaces and tuning gas-separation performance.

## 1. Introduction

The selective separation of CO_2_/CH_4_, CO_2_/N_2_, and CH_4_/N_2_ mixtures is essential for carbon capture, natural gas purification, biogas upgrading, and methane recovery from nitrogen-containing gas streams. CO_2_-rich flue gas from coal-fired power plants generally contains a large excess of N_2_, while CO_2_ and N_2_ impurities in biogas, coalbed methane, and low-grade natural gas reduce methane purity and energy density [1]. Therefore, developing porous adsorbents with high selectivity, sufficient adsorption capacity, good regenerability, and structural stability remains important for practical gas-separation processes.

Adsorptive separation technology is recognized as a promising carbon-capture and gas-separation approach using porous adsorbents, such as activated carbons [2], zeolites [3], and metal–organic frameworks (MOFs) [4]. Representative studies demonstrated that amine-modified zeolite composites can enhance CO_2_ adsorption and cyclic stability [5], while zeolite/MOF hybrid systems can improve CO_2_ uptake compared with the corresponding pristine components [6]. Among these porous materials, MOFs are particularly attractive because of their crystalline pore structures, tunable pore sizes, adjustable chemical environments, and abundant open metal sites [7]. However, many pristine MOFs still face practical limitations, including aggregation, moisture sensitivity, insufficient mechanical robustness, and performance decay during cyclic operation [8]. To overcome these limitations, pore-environment regulation, surface modification, and composite construction have been widely explored to improve MOF stability and separation performance [9].

Integrating MOFs with zeolites provides an effective route to combine the rigid inorganic framework, molecular-sieving channels, and ion-exchange capability of zeolites with the tunable coordination environment and open metal sites of MOFs [10]. Such zeolite–MOF composites can generate hierarchical porosity, interfacial adsorption regions, and modified diffusion pathways that are difficult to obtain from either component alone [11]. However, the controlled nucleation and growth of MOF species on zeolite surfaces remain challenging. Conventional synthetic strategies usually depend on externally supplied soluble metal ions, which may induce homogeneous nucleation in solution, generate separated MOF particles, and weaken control over the zeolite–MOF interfaces. Güemes et al. [12] showed that zeolite-derived metal species can participate in MOF formation at the zeolite interface, while Li et al. [13] demonstrated that insufficient heterogeneous nucleation sites can lead to fragmented and discontinuous MOF growth. Choi et al. [14] further reported that ion-exchange-induced crystallization can improve the interfacial compatibility of zeolite@MOF structures. These studies indicate that localized metal centers associated with porous supports can play a decisive role in directing MOF nucleation. Despite these advances, most reported zeolite–MOF composites are still discussed mainly in terms of final morphology, porosity, and adsorption performance, while the early-stage formation of MOF sol–gel species (primary structural units, abbreviated as PSUs) and their time-dependent interfacial evolution remain insufficiently understood. In particular, it remains unclear how exchangeable metal ions confined in zeolite frameworks coordinate with organic ligands, how the resulting metal-ligand species evolve during crystallization, and how this evolution regulates pore blockage, adsorption affinity, and molecular discrimination. Addressing these questions is important because the separation behavior of zeolite–MOF composites is governed not only by the presence of a MOF phase, but also by the accessibility of metal sites, the local coordination environment, the degree of interfacial ordering, and the diffusion resistance created during confined growth.

Clinoptilolite (CP), a HEU-type zeolite, contains interconnected 10-membered and 8-membered ring channels together with abundant exchangeable extra-framework cations [15]. These ion-exchange sites can provide stable anchoring positions for metal ions and may serve as localized metal reservoirs under suitable reaction conditions [16,17]. For example, the exchangeable Cu^2+^ species distributed in CPs were used to promote MOF-related interfacial construction, suggesting that pre-implanted metal sites can assist heterogeneous nucleation [18]. However, whether Mg^2+^-exchanged platelet CPs can supply sufficient localized Mg^2+^ centers to initiate and regulate the formation of Mg-MOF-74-related PSUs remains unclear. This question is particularly important because Mg^2+^ has strong coordination affinity toward oxygen-containing ligands but its availability, mobility, and coordination environment may be strongly affected by confinement within the CP framework.

Mg-MOF-74 is a representative MOF for low-pressure CO_2_ capture because of its high density of open Mg^2+^ sites and one-dimensional channels [19]. Nevertheless, bulk Mg-MOF-74 is prone to aggregation and suffers from limited water stability, which restricts its practical application. Du Bois et al. [20] revealed that Mg-MOF-74 formation involves ligand deprotonation, carboxylate coordination, and progressive Mg-O structural development, indicating that the Mg^2+^ supply mode and ligand-coordination kinetics are critical for regulating crystallization. Meanwhile, defect generation, unsaturated coordination environments, and partially developed MOF-74-type structures can influence gas adsorption through modified pore structures and local adsorption sites [21,22]. In zeolite-based systems, the organic-ligand modification and MOF-fragment decoration have also been shown to alter adsorption selectivity and diffusion behavior by regulating extra-framework cation environments, pore apertures, and molecular transport pathways [23]. These findings suggest that confined Mg^2+^-directed interfacial growth of Mg-MOF-74 PSUs on platelet CP (PCP) could provide a useful strategy for tuning CO_2_/CH_4_, CH_4_/N_2_, and CO_2_/N_2_ separation.

Herein, Mg-PCP was employed as a confined Mg^2+^ source, and 2,5-dihydroxyterephthalic acid (DHTA) was used as the organic ligand to induce the in situ interfacial growth of Mg-MOF-74-related PSUs. A series of PSU@Mg-PCP composites were prepared at different crystallization times to reveal the transition from disordered Mg-DHTA coordination species to locally ordered MOF-related interfacial subunits. Our previous study [24] employed direct interfacial growth of Mg-MOF-74 on CP surfaces, with emphasis on the final composite and its gas-separation performance. In contrast, the present work uses pre-exchanged Mg-PCP as a confined internal Mg^2+^ source, without introducing an external Mg salt during PSU growth, and focuses on the crystallization-time-dependent evolution of Mg-DHTA species toward Mg-MOF-74-related interfacial PSUs. This evolution is correlated with changes in local Mg-O coordination, pore accessibility, molecular transport, and gas-separation behavior. Thus, the present study extends the previous direct-growth approach to a confined-metal-source strategy for regulating early-stage interfacial PSU formation. Their structural and textural evolution was investigated using X-ray diffraction (XRD) and small-angle X-ray scattering (SAXS) patterns, Fourier transforms infrared (FT-IR), X-ray photoelectron spectroscopy (XPS) and extended X-ray absorption fine structure (EXAFS) spectra, scanning electron microscope (SEM) images, N_2_ sorption isotherms, and thermogravimetric (TG) analysis. In particular, the SAXS patterns were used to evaluate the surface fractal evolution of the composites, while Mg K-edge EXAFS provided local structural insight into the evolution of the Mg-O coordination environment during confined PSU growth. The adsorption and separation performances toward CO_2_/CH_4_, CH_4_/N_2_, and CO_2_/N_2_ were evaluated to establish the relationship between interfacial evolution and gas-separation behavior. Grand Canonical Monte Carlo (GCMC) simulations were further used to clarify the spatial distribution of CO_2_, CH_4_, and N_2_ and to identify the roles of Mg^2+^ sites, micropore blockage, and newly formed interfacial adsorption regions. This work provides mechanistic insight into confined-metal-source-driven MOF subunit growth on zeolite supports and offers a strategy for designing interface-regulated adsorbents for gas separation.

## 2. Results and Discussion

### 2.1. Characterizations of PSU@Mg-PCPs

**Figure 1:**
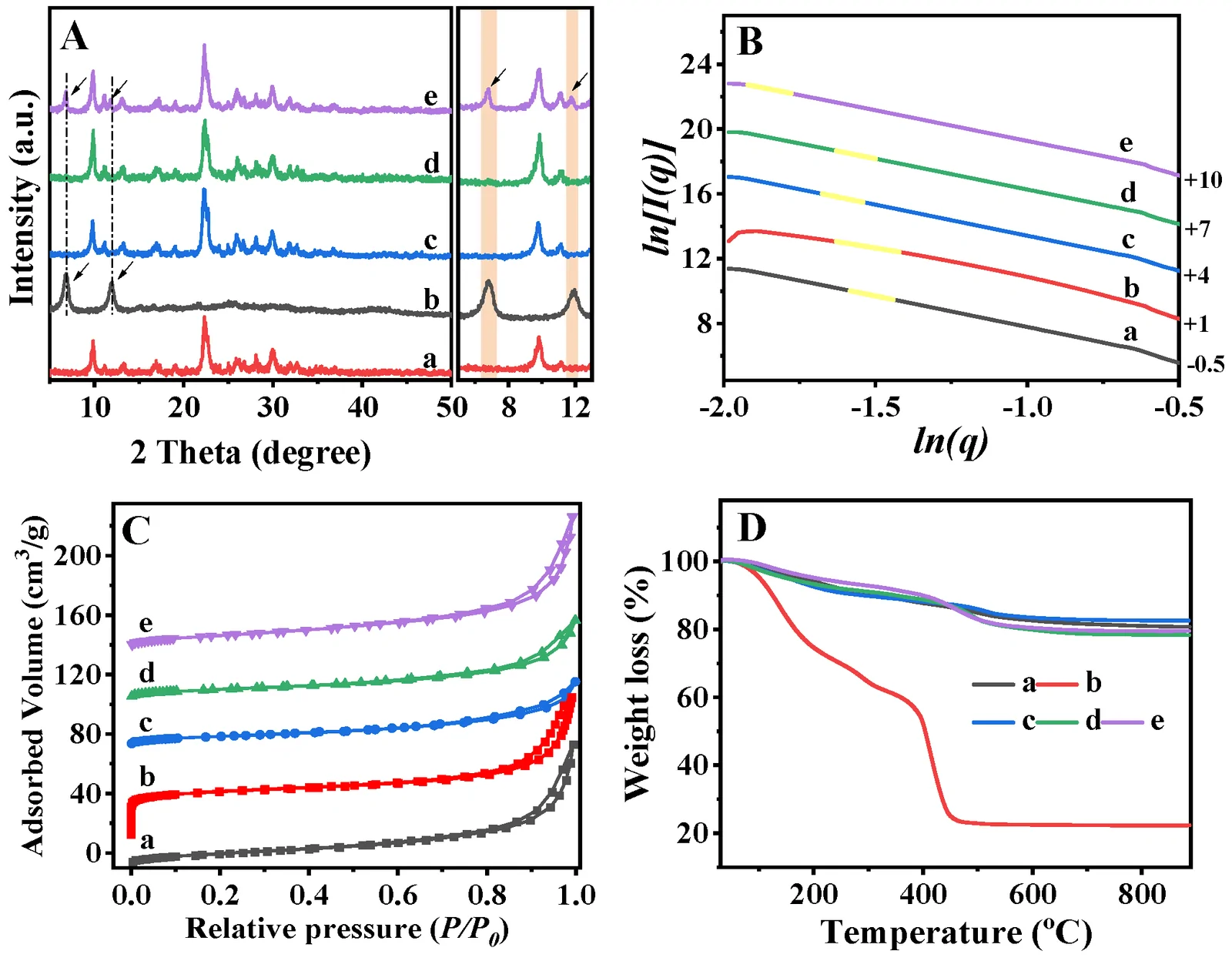
XRD (**A**) and vertically shifted SAXS (**B**) patterns, N_2_ adsorption–desorption isotherms at 77 K (**C**) and TG curves (**D**) of Mg−PCP (a), Mg−MOF−74 (b), PSU@Mg−PCP-10 (c), PSU@Mg−PCP−60 (d), and PSU@Mg−PCP−180 (e).

The crystalline structures of Mg-PCP, Mg-MOF-74, and PSU@Mg-PCP composites were examined by XRD patterns (Figure 1A). Mg-PCP (Figure 1A-a) exhibited characteristic HEU-type CP reflections indexed to the (020), (200), (111), (13-1), (131), (22-2), (42-2), (350), (530), and (061) planes, confirming that the HEU framework was retained after ion exchange and Mg^2+^ modification [25]. Compared with cetyltrimethylammonium bromide (CTAB)-PCP (Figure S1a in the Supporting Information (SI) section), PCP (Figure S1b) showed slightly weaker diffraction intensities, which can be attributed to partial CTA^+^ removal during ultrasonication and acid treatment, consistent with previous reports [26,27]. After NH_4_^+^ exchange (Figure S1c), the (020), (200), and (131) reflections shifted slightly to lower angles, indicating enlarged interplanar spacing due to Na^+^/K^+^ replacement by NH_4_^+^. After Mg^2+^ exchange (Figure 1A-a), the (200) reflection intensity was markedly weakened, suggesting increased disorder along the *a*-axis of the platelet framework. Mg-MOF-74 (Figure 1A-b) showed characteristic reflections at 2*θ* = 6.8° and 12.02°, consistent with reported Mg-MOF-74 structures [28,29,30].

For PSU@Mg-PCP composites (Figure 1A-c, -d, and -e), the HEU-type framework peaks remained dominant, indicating that the CP matrix was preserved during interfacial growth. Meanwhile, the CP-related (200) reflection intensity gradually weakened with increasing crystallization time, suggesting preferential formation of Mg-DHTA coordination species on the disordered platelet surfaces. Weak Mg-MOF-74-related reflections at 2θ = 6.8° and 12.02° became more evident for PSU@Mg-PCP-180 (Figure 1A-e), indicating the formation of limited, highly dispersed, or poorly crystallized Mg-MOF-74-related domains rather than a dominant bulk MOF phase. These results support a nucleation-confined growth process, in which extra-framework Mg^2+^ sites act as localized coordination centers for DHTA while extensive Mg-MOF-74 crystallization is restricted, similar to confined zeolite–MOF growth reported previously [31,32].

The SAXS patterns were further used to probe the interfacial fractal evolution of Mg-PCP and PSU@Mg-PCP composites (Figure 1B). The scattering profiles were fitted by a linear least-squares method in selected *q* regions, and the surface fractal dimension (*Ds*) value was calculated from the slope of the *ln[I(q)]*−”*ln(q)* plots. The fitting parameters are summarized in Table 1. All samples showed good linearity with slopes between −3 and −4, indicating typical surface-fractal characteristics. The *Ds* values of PSU@Mg-PCP-10, -60, and -180 (Figure 1B-c, -d, and -e) were 2.19, 2.23, and 2.35, respectively, slightly higher than that of Mg-PCP (2.16, as shown in Figure 1B-a), but lower than that of bulk Mg-MOF-74 (2.82, as shown in Figure 1B-b). This gradual increase in *Ds* values indicates progressive roughening and structural heterogenization of the Mg-PCP interface during confined Mg-DHTA coordination. At the early stage, limited DHTA coordination occurred on the Mg-PCP surfaces, causing only a slight increase in surface roughness. With extended crystallization, more Mg-DHTA coordination units accumulated at the surfaces and interfaces of Mg-PCP, forming a rougher and more heterogeneous interfacial layer. After 180 min, the further increase in *Ds* values suggests the evolution of more developed Mg-MOF-74-related PSUs; however, the value remained lower than that of bulk Mg-MOF-74, supporting interface-confined growth rather than independent MOF crystallization.

The porous characteristics of Mg-PCP and PSU@Mg-PCP composites were evaluated using N_2_ adsorption–desorption isotherms. As can be seen in Figure 1C, all samples showed a sharp N_2_ uptake at low relative pressure (*P/P*_0_ < 0.1), indicating the presence of micropores [24]. The H4-type hysteresis loops at high relative pressure (0.8 < *P/P*_0_ < 1.0) suggest the coexistence of slit-like mesopores or interparticle voids associated with the stacked platelet morphology [33]. The pore size distributions were mainly concentrated at 0.9–1.0 nm (Figure S2), further confirming the dominant microporous characteristics of these materials. The BET surface areas of PSU@Mg-PCP-10 (Figure 1C-c) and PSU@Mg-PCP-60 (Figure 1C-d) were 25.9 and 25.1 m^2^·g^−1^, respectively, close to that of Mg-PCP (26.9 m^2^·g^−1^, as shown in Figure 1C-a), but much lower than that of Mg-MOF-74 (802.7 m^2^·g^−1^, as shown in Figure 1C-b). This result indicates that bulk Mg-MOF-74 was not substantially formed at short crystallization times, consistent with the weak Mg-MOF-74-related XRD reflections (Figure 1A). PSU@Mg-PCP-180 (Figure 1C-e) showed a slightly increased surface area of 31.6 m^2^·g^−1^, which may be associated with further development of Mg-MOF-74-related interfacial domains. Meanwhile, the micropore volume decreased from 0.13 cm^3^·g^−1^ for Mg-PCP (Figure 1C-a) to 0.07–0.09 cm^3^·g^−1^ for PSU@Mg-PCP-10 (Figure 1C-c) and PSU@Mg-PCP-60 (Figure 1C-d), suggesting partial occupation or blockage of Mg-PCP micropores by interfacial Mg-DHTA coordination species during the early growth stage.

TG analysis was used to assess organic incorporation and thermal evolution (Figure 1D). Mg-PCP (Figure 1D-a) showed 19.2 wt% mass loss below 900 °C, mainly from physically adsorbed water at 30–200 °C and residual CTAB decomposition. Pure DHTA showed 99.3 wt% mass loss below 900 °C (Figure S3), indicating nearly complete organic decomposition. The detailed parameters are listed in Table S1. The mass loss increased from 17.5% for PSU@Mg-PCP-10 (Figure 1D-c) to 20.6% for PSU@Mg-PCP-180 (Figure 1D-e), evidencing progressive incorporation of DHTA-derived coordination species on Mg-PCP surfaces and interfaces. In contrast, Mg-MOF-74 (Figure 1D-b) showed much larger mass losses of 35.4% at 300 °C and 60.8% at 420 °C, confirming that PSU@Mg-PCP composites contained limited Mg-MOF-74-related domains rather than a dominant bulk MOF phase.

To further evaluate the incorporation of DHTA-derived species in the PSU@Mg-PCP composites, elemental analysis was performed. Mg-PCP exhibited a carbon content of 1.68 wt%, whereas the carbon contents of PSU@Mg-PCP-10, PSU@Mg-PCP-60, and PSU@Mg-PCP-180 were 1.99, 4.95, and 6.88 wt%, respectively. Using Mg-PCP as the baseline, the corresponding ΔC values were 0.31, 3.27, and 5.20 wt%, giving estimated DHTA-equivalent loadings of 0.64, 6.47, and 10.72 wt%, respectively. The progressive increase in DHTA-equivalent loading with crystallization time provides quantitative support for the increasing incorporation of DHTA-derived interfacial species during the evolution of the PSU@Mg-PCP composites.

Derivative TG profiles further clarify the Mg-DHTA structural evolution (Figure S3). The maximum weight-loss temperature of the physical mixture was close to that of DHTA but lower than those of PSU@Mg-PCP-10 and PSU@Mg-PCP-180 (Figure S3B-b and -d), while Mg-PCP showed a maximum at 100.6 °C (Figure S3B-a). This indicates enhanced thermal stability after Mg-O coordination at PCP surfaces/interfaces. The maximum mass-loss temperature decreased from 510 °C for PSU@Mg-PCP-10 (Figure S3B-b) to 461 °C for PSU@Mg-PCP-180 (Figure S3B-d), approaching Mg-MOF-74 (420 °C, Figure S3B-e). This shift suggests progressive evolution from disordered Mg-DHTA coordination networks toward Mg-MOF-74-related structural units, supporting nucleation-limited interfacial growth [34].

**Figure 2:**
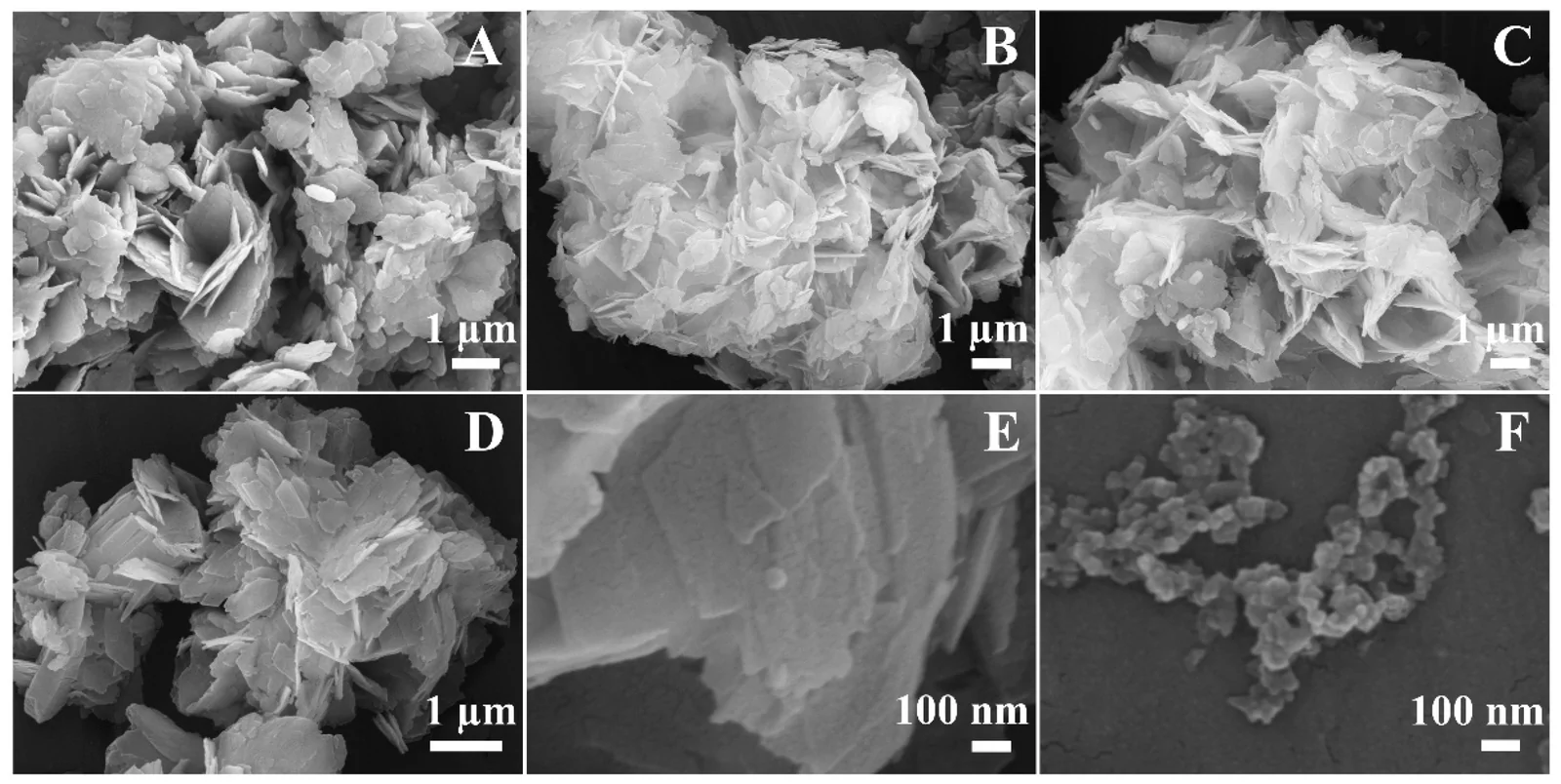
SEM images of Mg-PCP (**A**), PSU@Mg-PCP-10 (**B**), PSU@Mg-PCP-60 (**C**), PSU@Mg-PCP-180 (**D**,**E**), and Mg-MOF-74 (**F**).

The SEM images were used to examine the morphological evolution of Mg-PCP, PSU@Mg-PCP composites, and Mg-MOF-74 (Figure 2). Mg-PCP (Figure 2A) showed relatively uniform nanoflower-like particles assembled from stacked platelets, with sizes of approximately 1–3 μm. PSU@Mg-PCP-10 (Figure 2B), PSU@Mg-PCP-60 (Figure 2C), and PSU@Mg-PCP-180 (Figure 2D,E) retained similar platelet-stacking morphologies and particle sizes, indicating that the Mg-PCP architecture was largely preserved after interfacial Mg-DHTA coordination. In contrast, Mg-MOF-74 appeared as irregular aggregated nanoparticles of approximately 30–50 nm (Figure 2F). The PSU@Mg-PCP composites showed thinner nanosheet-like platelets than Mg-PCP, likely due to delamination during ultrasonication, acid washing, and solvent treatment. No obvious discrete Mg-MOF-74 particles were observed on PSU@Mg-PCP-10 (Figure 2B) or PSU@Mg-PCP-60 (Figure 2C), consistent with the weak Mg-MOF-74-related XRD reflections (Figure 1A-c and -d). For PSU@Mg-PCP-180 (Figure 2E), the irregular particles of approximately 40 nm appeared on the platelet surfaces, suggesting further development of Mg-MOF-74-related domains, consistent with the demonstrations of the XRD patterns (Figure 1A).

The SEM-EDS mappings further confirmed the elemental distribution in the PSU@Mg-PCPs (Figure S4), with the corresponding compositions summarized in Table S1. The major elements were uniformly distributed, indicating good dispersion of the interfacial coordination species. The C signal originated from DHTA-derived organic components, while Mg was associated with extra-framework Mg^2+^ species in CP and Mg-DHTA coordination sites. The Mg contents measured by EDS were lower than those determined by AAS measurement, likely because EDS reflected surface composition whereas AAS measurement provided bulk elemental information.

The FT-IR spectra of the samples are shown in Figure S5. For Mg-PCP (Figure S5-a), the band at 1211 cm^−1^ was assigned to the asymmetric stretching vibration of T-O-T (T = Si or Al), while the band at 793 cm^−1^ corresponded to O-T-O stretching. The peaks at 603 and 449 cm^−1^ were attributed to bending vibrations of external tetrahedral double rings and internal T-O bonds, respectively, and the band at 3627 cm^−1^ corresponded to -OH stretching [35,36]. For Mg-MOF-74 (Figure S5-d), the bands at 1206 and 1427 cm^−1^ were assigned to O-H and C=O vibrations, respectively, while the peak at 1588 cm^−1^ corresponded to asymmetric COO^−^ stretching [37]. The peak at 587 cm^−1^ was assigned to Mg-O vibration, consistent with previous reports [38,39]. PSU@Mg-PCP-10 (Figure S5-b) and PSU@Mg-PCP-180 (Figure S5-c) showed bands at 1427 and 1588 cm^−1^, indicating coordination between DHTA and Mg^2+^. In addition, the Mg-O vibration at 579 cm^−1^ further supported Mg^2+^ coordination on the CP surfaces and the formation of Mg-MOF-74-related Mg-DHTA species [40,41].

**Figure 3:**
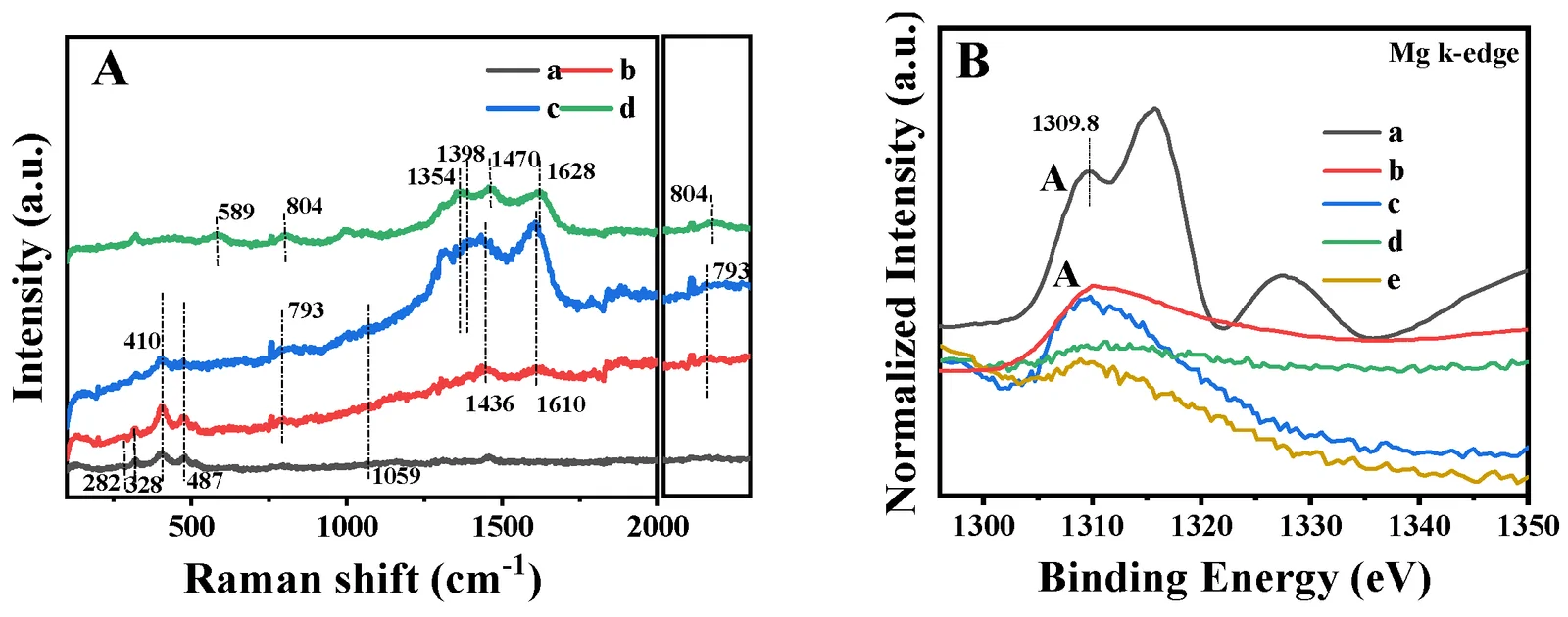
UV−Raman spectra (**A**) of Mg−PCP (a), PSU@Mg−PCP−10 (b), PSU@Mg−PCP−180 (c), and Mg−MOF−74 (d), and Mg K−edge EXAFS spectra (**B**) of MgO (a), Mg−MOF−74 (b), PSU@Mg−PCP−10 (c), PSU@Mg−PCP−60 (d), and PSU@Mg−PCP−180 (e).

The UV-Raman spectra were further used to probe the confined interfacial growth of Mg-DHTA species on PCP (Figure 3A). For Mg-PCP (Figure 3A-a), the peaks near 282 and 328 cm^−1^ were assigned to characteristic vibrations of 8-membered and 6- membered rings, respectively. The peak around 410 cm^−1^ was attributed to oxygen vibrations in Si-O-Al units within the CP framework. The band near 487 cm^−1^ was assigned to monomeric silicate species, while the peak around 1059 cm^−1^ corresponded to Si-O stretching in double four-membered rings. For Mg-MOF-74 (Figure 3A-d), the peak at 589 cm^−1^ was assigned to Mg-O vibration associated with coordination between Mg^2+^ and carboxyl groups. Compared with Mg-MOF-74, PSU@Mg-PCP-10 (Figure 3A-b) and PSU@Mg-PCP-180 (Figure 3A-c) showed an additional band at 793 cm^−1^, likely arising from a red shift in the 804 cm^−1^ band. This shift indicates a changed local Mg-O coordination environment in the composites, supporting DHTA-Mg^2+^ coordination and suggesting the formation of unsaturated Mg-MOF-74-related subunits during confined growth.

The local coordination environment around Mg was examined by Mg K-edge EXAFS using MgO as a reference (Figure 3B). The peak A features of the composites appeared at 1307–1313 eV, indicating Mg^2+^-containing local coordination environments. The systematic changes in peak position from PSU@Mg-PCP-10 (Figure 3B-c) to PSU@Mg-PCP-60 (Figure 3B-d) and PSU@Mg-PCP-180 (Figure 3B-e) suggested a crystallization-time-dependent evolution of the local Mg-O coordination environment. The corresponding Mg-O bond lengths were therefore derived from the empirical correlation reported in Ref. [42] and are listed in Table 1.

The peak A energy at the Mg K-edge is positively correlated with Mg-O bond length (*d_Mg-O_*), as described by Equation (1) [42]:

(1) $$y=-27.77x^{2}+123.60x+1174.16$$

where *y* is the peak A energy, *x* is *d_Mg-O_* expressed in Å, and the correlation coefficient is 0.986.

The estimated Mg-O bond length increased from 1.93 Å for PSU@Mg-PCP-10 to 1.94 Å for PSU@Mg-PCP-60 and 1.99 Å for PSU@Mg-PCP-180 (Table 1), progressively approaching that of Mg-MOF-74 (2.01 Å). This gradual increase indicates a continuous reconstruction of the local Mg coordination environment with prolonged crystallization. Because the estimated Mg-O bond lengths of the composites remained slightly shorter than that of Mg-MOF-74, their local Mg-DHTA coordination structures had not completely evolved into the coordination environment of crystalline Mg-MOF-74, supporting the presence of incompletely developed Mg-MOF-74-related interfacial structures.

The Mg-O bond valence was further evaluated using the bond-valence equation proposed by Brese and O’Keeffe [43]:

(2) $$\nu_{Mg\text{-}O}=exp\left(\frac{R_{Mg\text{-}O}-\;d_{Mg\text{-}O}}{b}\right)$$

where *R*_Mg-O_ is the Mg-O bond-valence parameter (1.693 Å), *b* is 0.37 Å, and *ν*_Mg-O_ is the Mg-O bond valence.

Considering the Mg^2+^ charge, the CN was estimated using Equation (3) [43]:

(3) $$\mathrm{CN}\;=\;\frac{2}{\nu_{Mg\text{-}O}}$$

A smaller bond-valence value corresponds to a larger calculated CN. As shown in Table 1, *ν*_Mg-O_ decreased from 0.53 to 0.51 and 0.45 for PSU@Mg-PCP-10, PSU@Mg-PCP-60, and PSU@Mg-PCP-180, respectively, while the calculated CN increased from 3.75 to 3.94 and 4.41. The progressive increase in CN toward the value calculated for Mg-MOF-74 (4.76) supported the gradual development of the local Mg-O coordination environment toward Mg-MOF-74-related structural units with increasing crystallization time. Overall, the Mg K-edge analysis and bond-valence results supported the time-dependent evolution of Mg-DHTA coordination species toward more developed Mg-MOF-74-related interfacial structures in the PSU@Mg-PCP composites.

**Figure 4:**
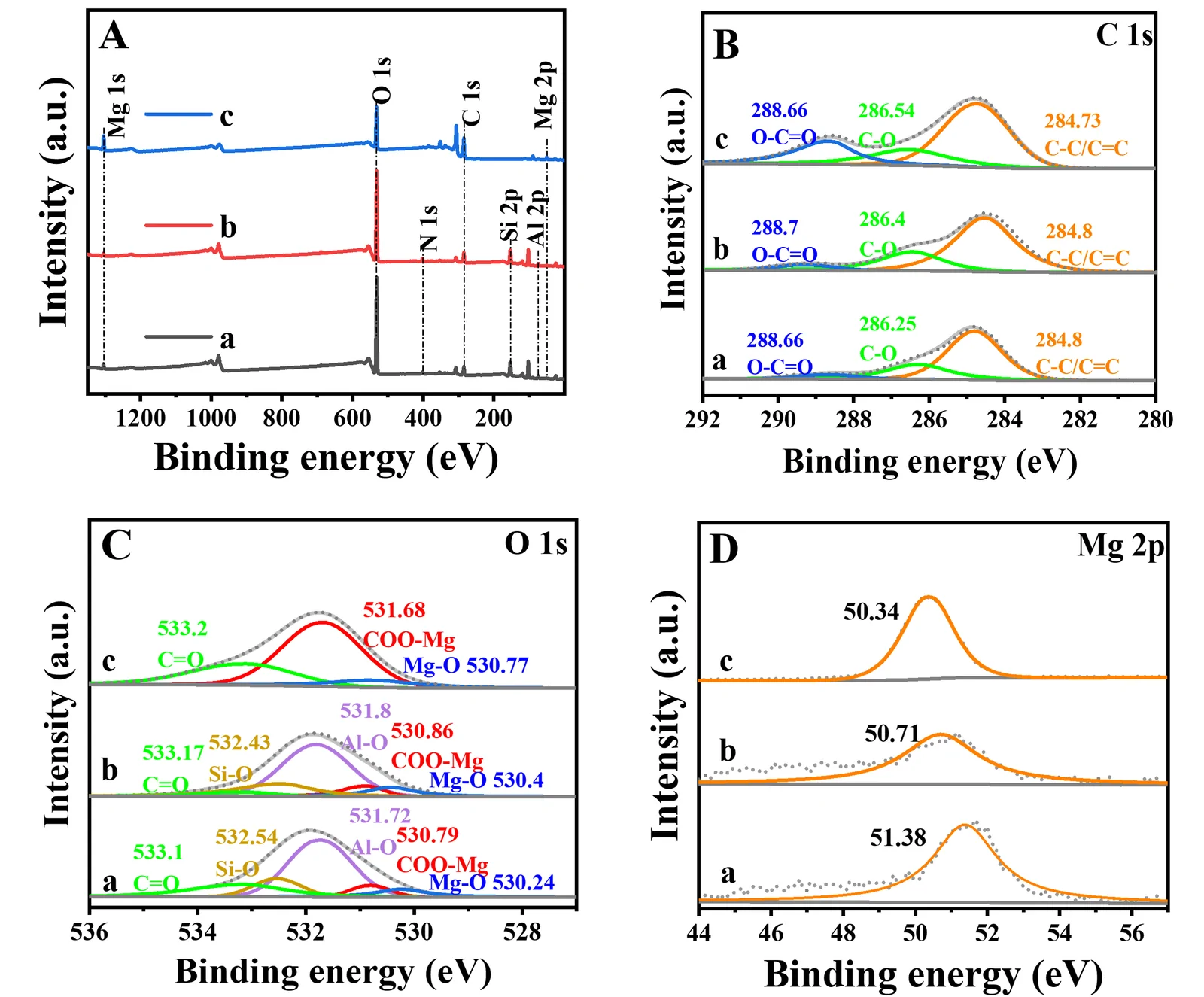
XPS spectra (**A**) and C1s (**B**), O1s (**C**), Mg2p (**D**) spectra of PSU@Mg-PCP-10 (a), PSU@Mg-PCP-180 (b), and Mg-MOF-74 (c).

The XPS spectra were used to examine surface chemical states and Mg-DHTA coordination (Figure 4). The survey spectra showed Mg 1s, Mg 2p, Si 2p, and Al 2p signals, while C 1s, O 1s, and N 1s mainly arose from residual CTAB and DHTA-derived species, confirming DHTA incorporation into Mg-PCP [44]. In the C 1s spectra, the peaks at 286.25 and 288.66 eV for PSU@Mg-PCP-10 (Figure 4B-a) were assigned to C-O and O-C=O groups of DHTA, respectively [45]. For PSU@Mg-PCP-180 (Figure 4B-b), the O-C=O peak shifted slightly to 288.70 eV, indicating a changed carboxyl electronic environment after Mg^2+^ coordination. In the O 1s spectrum of PSU@Mg-PCP-10 (Figure 4C-a), the peaks at 533.1, 530.79, and 530.24 eV were assigned to C=O, COO-Mg, and Mg-O species, respectively, confirming coordination between Mg^2+^ and DHTA oxygen atoms. For PSU@Mg-PCP-180 (Figure 4C-b), these peaks shifted to 533.1, 530.86, and 530.4 eV, respectively, indicating further evolution of the oxygen coordination environment with crystallization time. In contrast, the Si-O and Al-O-related signals remained nearly unchanged, suggesting preservation of the CP framework during interfacial growth [46]. The Mg 2p spectra further reveal time-dependent Mg coordination evolution (Figure 4D). The Mg 2p binding energy decreased from 51.38 eV for PSU@Mg-PCP-10 (Figure 4D-a) to 50.71 eV for PSU@Mg-PCP-180 (Figure 4D-b), indicating increased electron density around Mg after prolonged coordination. Together with the O 1s shifts, this supports electron redistribution between ligand oxygen atoms and Mg^2+^ during Mg-O coordination. However, the Mg 2p binding energy of PSU@Mg-PCP-180 remained higher than that of fully crystallized Mg-MOF-74 (50.34 eV), suggesting unsaturated Mg-O coordination structures rather than fully developed bulk Mg-MOF-74. The surface Mg contents from XPS spectra were 1.03% for PSU@Mg-PCP-10 and 0.93% for PSU@Mg-PCP-180, close to the AAS-derived bulk values of 1.06% and 1.11%, respectively, indicating uniform Mg^2+^ distribution without obvious surface segregation.

**Figure 5:**
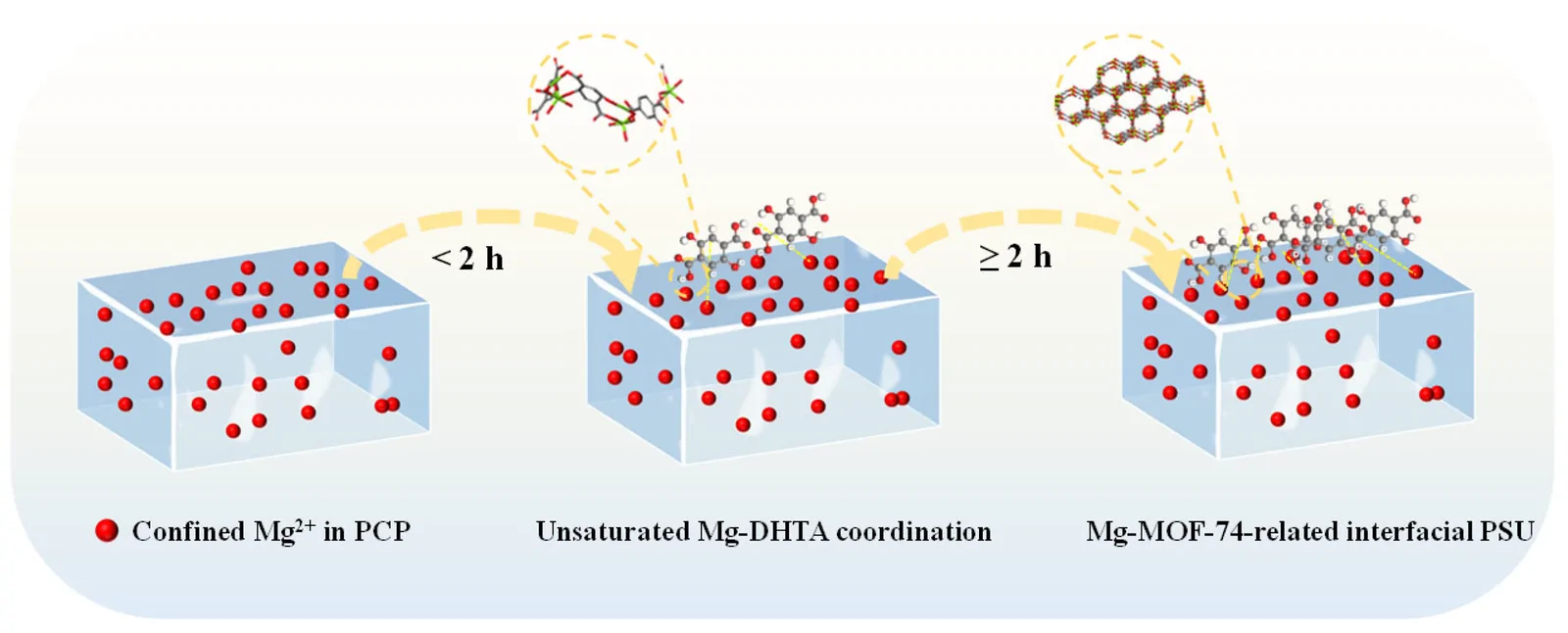
Schematic mechanism of the composite preparation.

The proposed interfacial formation pathway of the PSU@Mg-PCP composites is illustrated in Figure 5. After DHTA was introduced into the hydrothermal system, its carboxyl groups coordinated with extra-framework Mg^2+^ sites on Mg-PCP, forming interfacial Mg-O coordination bonds and immobilizing Mg^2+^ on the platelet surface. As the crystallization time increased from 10 min to 3 h, Mg-DHTA coordination structures progressively developed on the Mg-PCP surfaces and interfaces, generating Mg-DHTA sol–gel species and Mg-MOF-74-related subunits. The O-C=O, COO-Mg, and Mg-O signals in the C 1s and O 1s spectra confirmed Mg^2+^-DHTA coordination. With prolonged crystallization, the decrease in Mg 2p binding energy and increase in O 1s binding energy indicated enhanced Mg-O coordination interactions. However, the Mg-O structures remained unsaturated, leading to surface-accessible Mg-MOF-74-related subunits rather than fully crystallized bulk Mg-MOF-74. The consistent Mg contents obtained from XPS spectra and AAS measurements further indicate that no significant Mg^2+^ surface segregation occurred during synthesis. Taken together, these results indicate that PSU@Mg-PCP-x is more appropriately described as a zeolite-supported interfacial coordination composite, in which Mg-DHTA/Mg-MOF-74-related structural units are immobilized on Mg-PCP rather than being present as a simple physical mixture of Mg-PCP and Mg-MOF-74-related species.

### 2.2. Gas Adsorption and Separation Performances

**Figure 6:**
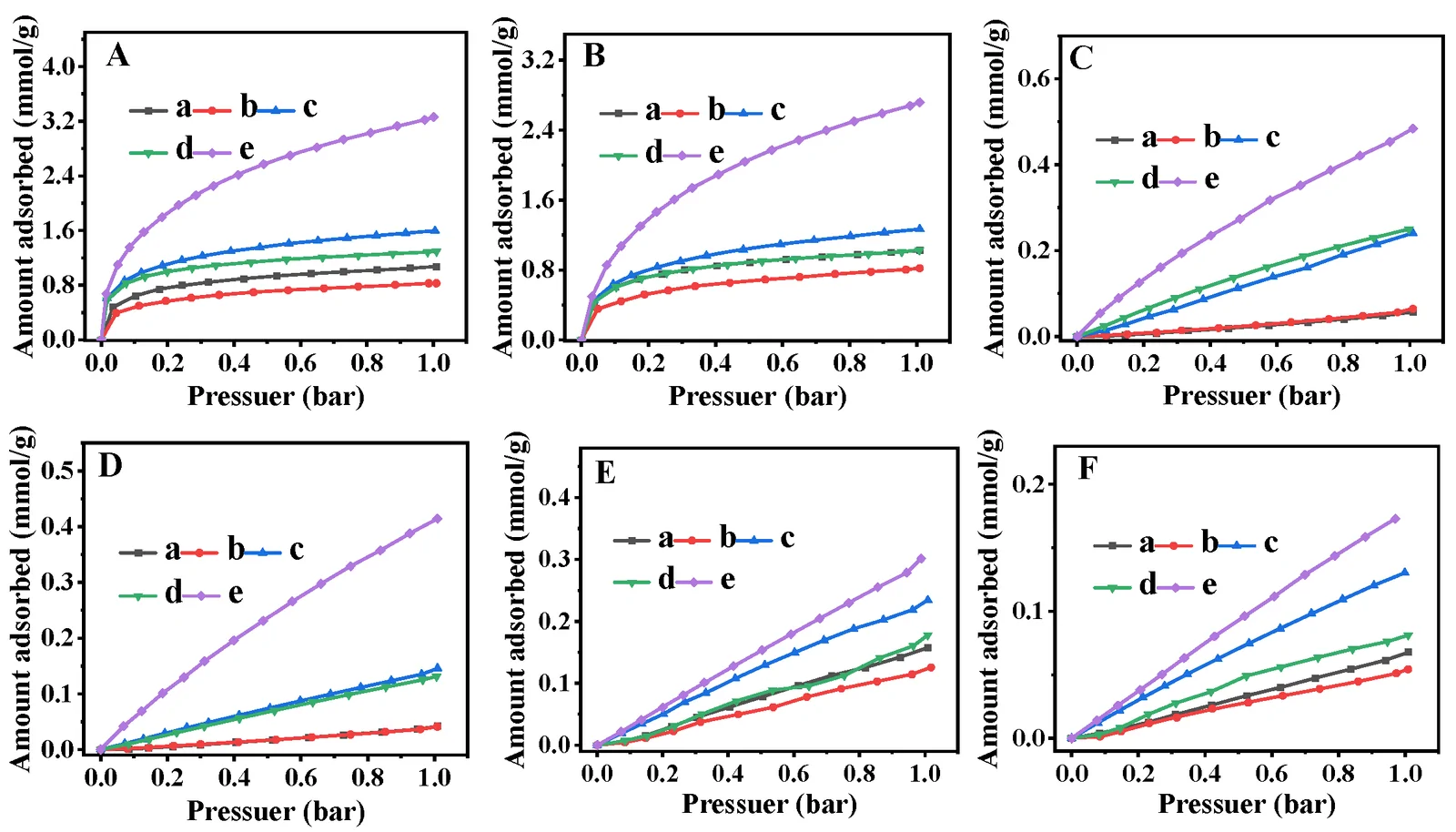
Adsorption isotherms of CO_2_ (**A**,**B**), CH_4_ (**C**,**D**), and N_2_ (**E**,**F**) on PSU@Mg−PCP−10 (a), −60 (b), and −180 (c), Mg−PCP (d), and Mg−MOF−74 (e) at 273 K (**A**,**C**,**E**) and 298 K (**B**,**D**,**F**).

Single-component adsorption isotherms of CO_2_, CH_4_, and N_2_ were measured at 273 and 298 K to evaluate the gas adsorption behavior of Mg-PCP, Mg-MOF-74, and PSU@Mg-PCP composites (Figure 6).

CO_2_ uptake increased rapidly at low pressure (*P* < 0.1) and then gradually at higher pressure, indicating preferential adsorption at high-affinity sites. At 273 K, Mg-PCP (Figure 6A-d) showed a CO_2_ uptake of 1.29 mmol·g^−1^, higher than PSU@Mg-PCP-10 (1.07 mmol·g^−1^, Figure 6A-a) and PSU@Mg-PCP-60 (0.83 mmol·g^−1^, Figure 6A-b), but lower than PSU@Mg-PCP-180 (1.60 mmol·g^−1^, Figure 6A-c). At 298 K, the CO_2_ uptakes of PSU@Mg-PCP-10 (Figure 6B-a), PSU@Mg-PCP-60 (Figure 6B-b), and PSU@Mg-PCP-180 (Figure 6B-c) were 1.02, 0.82, and 1.27 mmol·g^−1^, respectively. All samples showed higher CO_2_ uptake at 273 K than at 298 K, consistent with physisorption-dominated adsorption.

As can be seen in Figure 6C, CH_4_ and N_2_ uptakes were much lower than CO_2_ uptake. At 273 K, PSU@Mg-PCP-10 (Figure 6C-a) and PSU@Mg-PCP-60 (Figure 6C-b) showed CH_4_ uptakes of 0.06 and 0.07·mmol·g^−1^, respectively, lower than Mg-PCP (0.25 mmol·g^−1^, Figure 6C-d). The N_2_ uptakes were 0.16 mmol·g^−1^ for PSU@Mg-PCP-10 (Figure 6E-a) and 0.13 mmol·g^−1^ for PSU@Mg-PCP-60 (Figure 6E-b), also lower than Mg-PCP (0.18 mmol·g^−1^, Figure 6E-d). These results indicate that early Mg-DHTA/PSU formation suppresses CH_4_ and N_2_ adsorption more strongly than CO_2_ adsorption.

The adsorption trend reflects crystallization-time-dependent pore regulation. At short crystallization time, limited Mg-DHTA species formed on Mg-PCP. After 60 min, increased interfacial Mg-DHTA/PSU species partially occupied or blocked Mg-PCP micropores, reducing gas uptake. After 180 min, more developed Mg-MOF-74-related interfacial domains generated additional adsorption sites, improving gas affinity, especially for CO_2_. Thus, the adsorption behavior is controlled by the balance between micropore blockage and formation of CO_2_-affinitive interfacial domains.

Apparent separation factors for CO_2_/CH_4_, CO_2_/N_2_, and CH_4_/N_2_ were calculated from the fitted single-component adsorption isotherms using the Freundlich-Langmuir (F-L) model. These values are used here as comparative adsorption metrics and are not interpreted as competitive mixture selectivities.

(4) $$Q=\frac{q_{s}KC^{n}}{1+{KC}^{n}}$$

where *Q* is the adsorbed amount (mmol·g^−1^), *q_s_* is the saturation adsorption capacity (mmol·g^−1^), *K* and *n* are F-L constants, and *C* is the relative pressure.

As shown in Figure S6A,B, the apparent CO_2_/CH_4_ separation factors decreased with increasing pressure. PSU@Mg-PCP-10 showed the highest apparent CO_2_/CH_4_ separation factors of 18.79 at 273 K and 24.23 at 298 K, higher than Mg-PCP (5.14 and 7.83), PSU@Mg-PCP-60 (12.65 and 20.08), and PSU@Mg-PCP-180 (6.63 and 8.67). This indicates that short-time PSU formation increases the relative preference for CO_2_ over CH_4_ under the single-component adsorption conditions, likely through the introduction of CO_2_-affinitive Mg-DHTA sites and restricted CH_4_ uptake. For CO_2_/N_2_ separation, the composites showed a non-monotonic crystallization-time dependence (Figure S6C,D). At 273 K, PSU@Mg-PCP-10 showed an apparent CO_2_/N_2_ separation factor of 6.76, slightly higher than PSU@Mg-PCP-60 (6.51) and higher than Mg-PCP and PSU@Mg-PCP-180 (both 5.67). At 298 K, PSU@Mg-PCP-10 and PSU@Mg-PCP-60 showed an apparent CO_2_/N_2_ separation factors of 15.01 and 15.22, respectively, both higher than Mg-PCP and PSU@Mg-PCP-180 (both 9.01). These results further indicate that moderate interfacial Mg-DHTA/PSU formation increases the relative adsorption preference for CO_2_ over N_2_ under the single-component conditions.

For CH_4_/N_2_ separation, the apparent separation factors of PSU@Mg-PCP composites increased with crystallization time but remained lower than those of Mg-PCP and Mg-MOF-74 (Figure S6E,F). At 273 and 298 K, PSU@Mg-PCP-10 showed apparent CH_4_/N_2_ separation factors of 0.35 and 0.60, respectively, lower than Mg-PCP (1.51 and 1.61), Mg-MOF-74 (1.57 and 2.32), PSU@Mg-PCP-60 (0.49 and 0.73), and PSU@Mg-PCP-180 (1.03 and 1.08). Therefore, the single-component adsorption results indicate that PSU formation mainly enhances the relative CO_2_ adsorption preference rather than CH_4_/N_2_ discrimination.

To further evaluate competitive adsorption, IAST predictions were performed using the fitted single-component CO_2_ and CH_4_ adsorption isotherms at 298 K. For an equimolar CO_2_/CH_4_ mixture at 1 bar, the IAST-predicted selectivities were 52.04, 43.15, 52.45, 56.21, and 62.34 for Mg-PCP, Mg-MOF-74, PSU@Mg-PCP-10, PSU@Mg-PCP-60, and PSU@Mg-PCP-180, respectively. These results indicate preferential CO_2_ adsorption under the competitive conditions represented by IAST. Notably, the IAST trend differs from that obtained from the apparent separation factors based on the single-component isotherms, further demonstrating that the latter should be regarded as comparative adsorption metrics rather than direct competitive-mixture selectivities.

The isosteric heat of adsorption (*Q_st_*) was calculated using the virial Equations:

(5) $$\ln\left(\frac{p}{n}\right)=\frac{1}{T}\sum_{j=0}^{L}a_{j}n^{j}+\sum_{j=0}^{m}b_{j}n^{j}$$

(6) $$Q_{st}\left(n\right)=-R\sum_{j=0}^{L}a_{j}n^{j}$$

where *a* and *b* are virial coefficients, *n* is the adsorbed amount at pressure *p*, *l* and *m* are the equation orders, and *R* is the gas constant (8.314 J·mol^−1^·K^−1^).

The *Q_st_* curves of CO_2_, CH_4_, and N_2_ for Mg-PCP and PSU@Mg-PCP composites are shown in Figure S7, and the values are summarized in Table S2. Most *Q_st_* values were below 40 kJ·mol^−1^, indicating predominantly physisorption-based interactions, except CO_2_ on PSU@Mg-PCP-180, which reached 40.48 kJ·mol^−1^. The higher CO_2_ *Q_st_* than CH_4_ and N_2_ indicates stronger CO_2_-adsorbent interactions, consistent with the adsorption isotherms.

**Figure 7:**
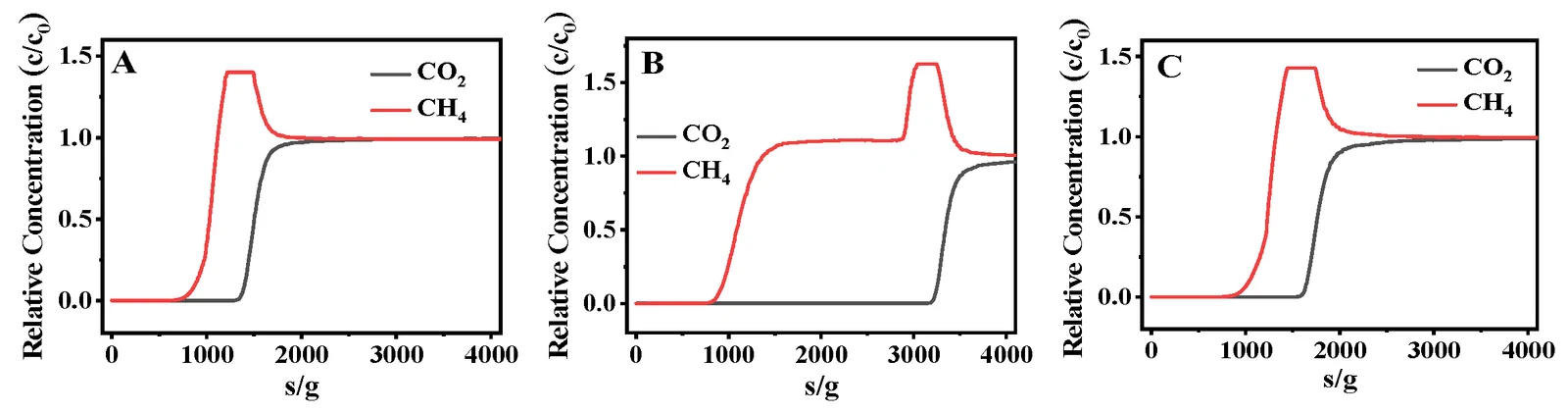
Breakthrough curves of CO_2_/CH_4_ mixed gas (50/50, *v*/*v*) at 298 K over Mg-PCP (**A**), Mg-MOF-74 (**B**), and PSU@Mg-PCP-10 (**C**).

Dynamic breakthrough experiments were conducted using a CO_2_/CH_4_ mixture (50/50, *v*/*v*) at 298 K (Figure 7). CH_4_ broke through earlier than CO_2_ for all samples, confirming stronger CO_2_ retention. The CO_2_ breakthrough time of PSU@Mg-PCP-10 (Figure 7C) was longer than Mg-PCP (Figure 7A) but shorter than Mg-MOF-74 (Figure 7B), while CH_4_ breakthrough times were similar. These results provide direct experimental validation of competitive CO_2_/CH_4_ separation and confirm the improved dynamic separation performance of PSU@Mg-PCP-10 relative to Mg-PCP.

**Figure 8:**
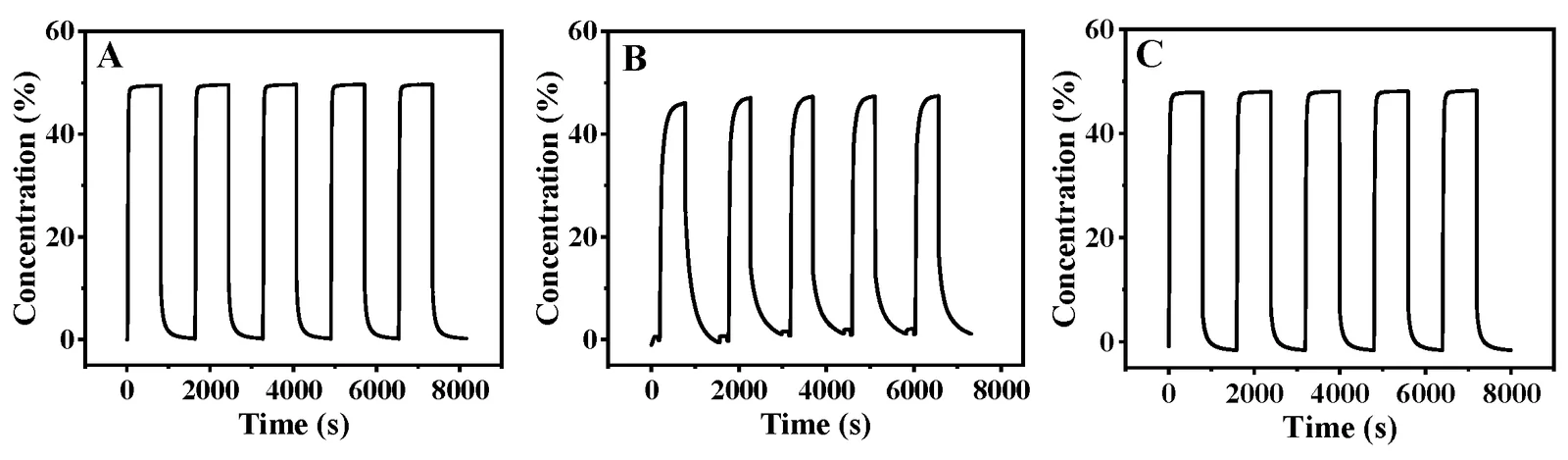
CO_2_ adsorption–desorption cycling performances of Mg-PCP (**A**), Mg-MOF-74 (**B**), and PSU@Mg-PCP-10 (**C**) at 298 K.

The CO_2_ adsorption stability of Mg-PCP, Mg-MOF-74, and PSU@Mg-PCP-10 was evaluated over five adsorption–desorption cycles at 298 K and 1 bar (Figure 8). After repeated He-purge regeneration for 30 min, the CO_2_ adsorption capacities remained nearly unchanged, indicating good cycling stability and reversibility. PSU@Mg-PCP-10 showed more stable cyclic performance than Mg-PCP, suggesting improved reusability after introducing Mg-MOF-74-related subunits.

### 2.3. GCMC Simulations

The optimized Mg-PCP, Mg-MOF-74, disordered PSU@Mg-PCP, and ordered PSU@Mg-PCP models are shown in Figure S8. Single-component CO_2_, CH_4_, and N_2_ isotherms were simulated at 298 K over 1–100 kPa (Figure S9). CO_2_ uptake increased rapidly below 0.1 bar and then gradually at higher pressures (Figure S9A). Mg-PCP showed a CO_2_ uptake of 4.71 mmol·g^−1^ (Figure S9A-a), lower than ordered PSU@Mg-PCP (10.82 mmol·g^−1^, Figure S9A-d) and Mg-MOF-74 (12.78 mmol·g^−1^, Figure S9A-b), but higher than disordered PSU@Mg-PCP (3.89 mmol·g^−1^, Figure S9A-c). This trend indicates that the disordered PSUs block Mg-PCP micropores, whereas the ordered PSUs create interfacial adsorption sites that promote CO_2_ uptake. CH_4_ and N_2_ showed more linear isotherms with lower uptakes (Figure S9B,C), consistent with weaker adsorption affinity and larger kinetic diameters. The simulated trends qualitatively agree with the experimental adsorption behavior; therefore, the computational models are used primarily to interpret relative structure-adsorption relationships and adsorption mechanisms rather than to quantitatively reproduce the experimental adsorption capacities.

Binary gas diffusion was also simulated to clarify the separation mechanism (Figure S10 and Table S3). Kinetic selectivity was defined as *S_kinetic_* = *D_A_/D_B_*. According to Henrique et al.’ report [47], greater deviation from 1 indicates stronger kinetic separation potential. For CO_2_/CH_4_ (as shown in Table S3), the kinetic selectivities were 0.4118, 0.2084, and 0.0294 for Mg-PCP, disordered PSU@Mg-PCP, and ordered PSU@Mg-PCP, respectively, showing slower CO_2_ diffusion relative to CH_4_ after PSU formation. A similar trend was also observed for CO_2_/N_2_, with selectivities below 1 for both PSU@Mg-PCP models. For CH_4_/N_2_, disordered PSU@Mg-PCP showed selectivity below 1, whereas ordered PSU@Mg-PCP exceeded 1, indicating faster CH_4_ diffusion after PSU ordering. Thus, PSU-induced micropore restriction and structural ordering regulate molecular diffusion. Based on the simulated isotherms (Figure S9), CO_2_/CH_4_ adsorption selectivities increased from 5.54 for Mg-PCP to 13.4 and 26.4 for disordered and ordered PSU@Mg-PCP, respectively. This increase suggests that CO_2_ separation is mainly governed by thermodynamic adsorption preference, while PSU-induced micropore restriction further modulates diffusion.

**Figure 9:**
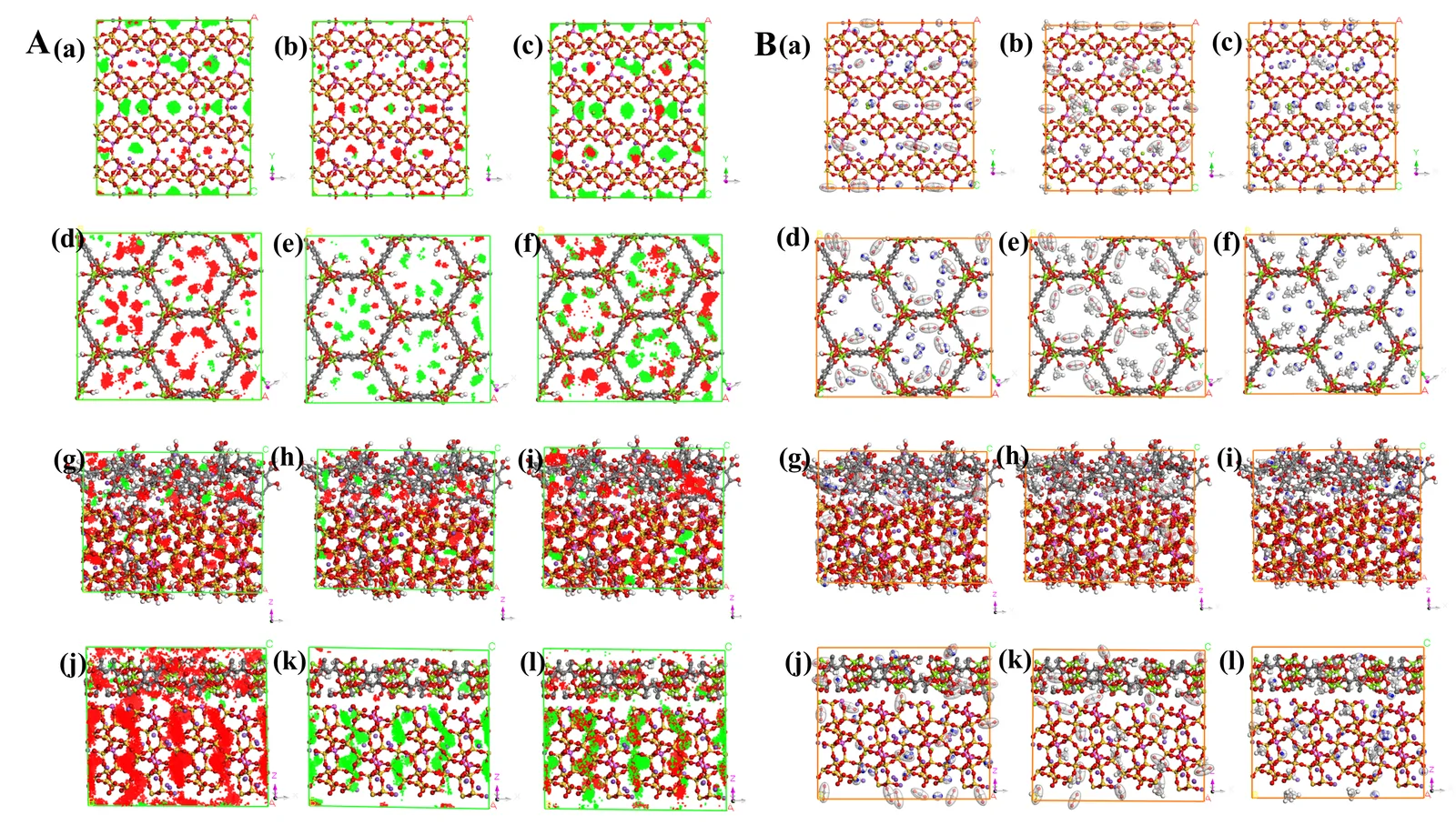
Simulated adsorption-density distributions (**A**) and adsorption sites (**B**) of binary gas mixtures at 298 K. CO_2_/N_2_ (**a**,**d**,**g**,**j**), CO_2_/CH_4_ (**b**,**e**,**h**,**k**), and CH_4_/N_2_ (**c**,**f**,**i**,**l**) in Mg-PCP (**a**,**b**,**c**), Mg-MOF-74 (**d**,**e**,**f**), disordered PSU@Mg-PCP (**g**,**h**,**i**), and ordered PSU@Mg-PCP (**j**,**k**,**l**).

The adsorption-density distributions and adsorption sites further reveal the molecular origin of separation (Figure 9). CO_2_ in disordered PSU@Mg-PCP (Figure 9A(h)) and ordered PSU@Mg-PCP (Figure 9A(k)) was mainly concentrated around MOF-related subunits and Mg-PCP interfaces, whereas CH_4_ and N_2_ were distributed near the matrix surface and micropore edges. The adsorption-site maps (Figure 9B(g,h,j,k)) show that blocked fragments restrict internal micropore access and shift adsorption toward PSU@Mg-PCP surfaces. Because CH_4_ and N_2_ interact more weakly and have larger kinetic diameters, their adsorption is more strongly inhibited by pore restriction. In contrast, CO_2_ remains preferentially adsorbed at original Mg^2+^ sites and newly formed interfacial regions [48]. Therefore, PSU-induced pore restriction and interfacial Mg^2+^-containing adsorption domains jointly account for the improved CO_2_-selective separation behavior.

The *IE* values between different CPs and gases were calculated using the Energy task in the Forcite module. As shown in Figure S11, the order of the absolute *IE* values of each kind of CP toward the gases was CO_2_ >> CH_4_ ≥ N_2_. This result could be well explained as follows: CO_2_ has a relatively large quadrupole moment and polarizability, and thus enables stronger electrostatic interactions with open metal sites or oxygen-containing functional groups, while CH_4_ and N_2_ mainly rely on weaker van der Waals forces [49]. Specifically, Mg-MOF-74 had the largest absolute *IE* value for CO_2_ (31.04 kcal·mol^−1^) due to its high density of the open metal sites, while its adsorption affinity for CH_4_ (4.42 kcal·mol^−1^) and N_2_ (2.30 kcal·mol^−1^) was the weakest, consistent with the reported results [50]. The *IE* values of Mg-LCP and ordered PSU@Mg-PCP were about −19.7 kcal·mol^−1^ for CO_2_, −4.7 to −5.4 kcal·mol^−1^ for CH_4_, and about −3.2 to −3.9 kcal·mol^−1^ for N_2_, indicating that both of them maintained a strong CO_2_ binding capacity. These simulated results suggested that the locally ordered structures of Mg-LCP and ordered PSU@Mg-PCP effectively preserved the micropore integrity and the accessibility of active sites [51]. However, the absolute *IE* value of disordered PSU@Mg-PCP was around 11.14 kcal·mol^−1^ for CO_2_, lower than that of Mg-LCP and ordered PSU@Mg-PCP. One of the probable reasons was due to the micropore blockages caused by the disordered PSU structures, thereby reducing the utilization efficiency of the adsorption sites. Obviously, these demonstrations implied that the well-ordered micropore structures were conducive to providing more accessible sites and therefore facilitate effective gas molecule adsorptions.

## 3. Conclusions

In present work, a confined metal-source strategy was developed to construct PSU@Mg-PCP composites using Mg-CP as both the porous support and internal Mg source. Without adding external magnesium salts, DHTA coordinated with extra-framework Mg^2+^ sites on Mg-PCP, inducing the in situ formation of Mg-MOF-74-related interfacial subunits. XRD, SEM, SAXS, XPS, and Mg K-edge XANES characterizations revealed a crystallization-time-dependent evolution from disordered Mg-DHTA coordination species to more ordered, unsaturated Mg-MOF-74-related interfacial domains. Increasing the hydrothermal crystallization time from 10 to 180 min raised the surface fractal dimension from 2.19 to 2.35. Meanwhile, the estimated Mg-O bond length increased from 1.93 to 1.99 Å and the calculated CN increased from 3.75 to 4.41 with increasing crystallization time, progressively approaching the corresponding values of Mg-MOF-74 and supporting the gradual development of Mg-MOF-74-related local coordination structures.

Gas adsorption results revealed a clear structure-performance relationship. PSU@Mg-PCP-10 showed high apparent CO_2_/CH_4_ and CO_2_/N_2_ separation factors derived from the single-component adsorption data, reaching 24.23 and 15.01 at 298 K, respectively, higher than those of Mg-PCP. In contrast, PSU@Mg-PCP-180 exhibited the highest CO_2_ uptake, reaching 1.60 mmol·g^−1^ at 273 K and 1.27 mmol·g^−1^ at 298 K. This difference indicates that short-time PSU formation enhances the relative CO_2_ adsorption preference under the single-component conditions through partial pore restriction and CO_2_-affinitive sites, whereas prolonged crystallization enhances CO_2_ capacity by generating more developed interfacial adsorption domains. Breakthrough experiments further verified preferential CO_2_ retention over CH_4_, and adsorption–desorption cycling demonstrated good regeneration stability.

GCMC simulations supported the experimental results by showing that PSU-induced micropore restriction suppresses CH_4_ and N_2_ adsorption, while CO_2_ preferentially binds to Mg^2+^ sites and newly formed interfacial regions. Overall, this study demonstrates an interfacial coordination strategy for constructing zeolite–MOF composites with tunable adsorption domains and provides mechanistic insight into confined MOF growth on porous aluminosilicate supports.

## 4. Materials and Methods

### 4.1. Materials

NaOH (99 wt%), KOH (96 wt%), Al(OH)_3_ (82 wt%), NH_4_Cl (99.5 wt%), HNO_3_ (68.0 wt%), and ethanol were supplied by Tianjin Fuchen Chemical Reagent Co., Ltd, Tianjin, China. Mg(NO_3_)_2_·6H_2_O and MgCl_2_ were provided by Beijing Yili Fine Chemicals Co., Ltd, Tianjin, China. CTAB (99%) was purchased from Bailingwei Technology Beijing Co., Ltd, Beijing, China. N, N-Dimethylformamide (DMF), methanol and ammonia solution (NH_3_·H_2_O, mass fraction 25–28%) were obtained from Tianjin Damao Chemical Reagent Co., Ltd, Tianjin, China. DHTA was purchased from Macklin Shandong Keyuan Biochemical Co., Ltd, Shandong, China. Aqueous colloidal silica sol (1.2 g mL^−1^, 30 wt% SiO_2_) was obtained from Qingdao Ocean Chemical Plant. Deionized water with a resistivity of 18.25 MΩ cm at 298 K was prepared in the laboratory. Natural CP (400 mesh) was used as the seed. All chemicals were of analytical grade and used without further purification.

### 4.2. Preparation of PCP and Mg-PCP

PCP was prepared according to our previous reports with minor modification [16,17]. Briefly, appropriate amounts of NaOH, KOH, Al(OH)_3_, and CTAB were mixed with 10 mL of deionized water in a 50 mL Teflon-lined autoclave. The mixture was stirred in an oil bath at 150 °C for 3 h. After cooling to room temperature, 8.8 mL of silica sol and 15 mL of deionized water were slowly added. Subsequently, 0.3 g of natural CP seed was dispersed into the mixture, followed by stirring at room temperature for 2 h. The resulting homogeneous suspension was transferred into the autoclave and crystallized at 140 °C for 4 days. The product was filtered, washed thoroughly with deionized water, and dried at 120 °C overnight. The obtained white powder was denoted as CTAB-PCP.

To remove CTAB and obtain PCP, 0.5 g of CTAB-PCP was dispersed in 60 mL of ethanol and sonicated at 50 W and 60 kHz for 6 h. The solid was collected by centrifugation and treated with 10 mL of 0.05 mol L^−1^ HNO_3_ under magnetic stirring at room temperature for 1 h. After centrifugation, the sample was washed with deionized water until the filtrate reached neutral pH. The obtained solid was dried at 120 °C overnight and denoted as PCP.

For NH_4_^+^ exchange, 1 g of PCP was added to 100 mL of 1 mol L^−1^ NH_4_Cl solution and stirred at 80 °C for 2 h. The product was filtered, washed, and dried. This exchange procedure was repeated 10 times to obtain NH_4_-PCP. Subsequently, 1 g of NH_4_-PCP was added to 200 mL of 0.06 mol L^−1^ MgCl_2_ solution and stirred at 80 °C for 2 h. The Mg^2+^-exchange process was repeated six times. The final product was filtered, washed thoroughly with deionized water, and dried at 120 °C overnight to obtain Mg-PCP.

### 4.3. Synthesis of PSU@Mg-PCP-x Composites

PSU@Mg-PCP-x composites were prepared through the in situ interfacial growth of Mg-MOF-74-related PSUs on Mg-PCP. Typically, 0.3 g of Mg-PCP was dispersed in 30 mL of DMF, while 23 mg of DHTA was dissolved in 5 mL of deionized water. The two solutions were mixed, followed by the addition of 0.5 mL of NH_3_·H_2_O. The mixture was transferred into an 80 mL Teflon-lined autoclave and reacted at 140 °C for 10, 60, or 180 min. After cooling naturally to room temperature, the products were collected by centrifugation, washed with DMF and methanol, soaked in methanol for 7 days with solvent replacement every 6 h, and dried at 120 °C for 12 h. The resulting composites were denoted as PSU@Mg-PCP-x, where x represents the crystallization time. Structurally, PSU@Mg-PCP-x is a zeolite-supported interfacial coordination composite containing Mg-DHTA/Mg-MOF-74-related PSUs immobilized on Mg^2+^-exchanged PCP rather than a simple physical mixture. The preparation process is illustrated in Scheme S1 of the Supporting Information.

### 4.4. Characterizations

The XRD patterns were obtained using a Beijing Purkinje General Instrument Co. Ltd, Beijing, China. XD-6 X-ray diffractometer with Cu Kα radiation (λ = 0.154056 nm) and a step size of 4 °·min^−1^ in a scan range of 5–50 ° (2*θ*). The FT-IR spectra were performed using a Shimadzu IR Prestige-21 spectrophotometer with KBr pellets in the range of 400–4000 cm^−1^, Shimadzu Corporation, Kyoto, Japan. The TG analysis was performed on a PerkinElmer Pyris I instrument under air from 25 to 900 °C at a heating rate of 10 °C min^−1^, Waltham, United States. XPS analysis was conducted using a Thermo Fisher K-Alpha Plus instrument, Waltham, United States. Metal-ion contents were determined by AAS using a Hitachi ZA3300 system, Tokyo, Japan. The elemental analysis was performed using elemental analysis instrument (Vario MICRO, Hanau, Germany). N_2_ adsorption–desorption isotherms were measured using a JW-BK300 instrument, Beijing, China. Before analysis, the samples were degassed under high vacuum at 120 °C for 3 h. The specific surface area was calculated using the BET method, and the pore volume was estimated from the adsorption amount at a relative pressure of 0.99. Pore size distributions were calculated using the Horvath Kawazoe model based on the adsorption branch of the isotherms. The material morphology was observed using a field emission scanning electron microscope (FE-SEM, Hitachi S-4800, Tokyo, Japan) at an accelerating voltage of 15.0 kV. Structural information of the material was obtained with a fully automatic laser confocal Raman spectrometer (Finder 930, Beijing, China). The laser source had a wavelength of 325 nm, and the laser power at the sample was maintained at 5.0 mW.

Mg K-edge EXAFS measurements were performed at the 1W2B beamline of the Beijing Synchrotron Radiation Facility [52]. The beamline optics included a Rh-coated collimating mirror with a grazing incidence angle of 3.6 mrad, a Si double-crystal monochromator (Δ*E/E* ≈ 2 × 10^−4^, 4.5–20 keV, >10^9^ photons s^−1^), and a Rh-coated toroidal focusing mirror, producing an X-ray spot of 1.74 mm × 0.73 mm. MgO was used as the reference sample.

The SAXS patterns were conducted at the 1W2B beamline of the Beijing Synchrotron Radiation Facility using synchrotron radiation [53]. The X-ray wavelength was 0.154 nm. A Pilatus 3S 1M detector (DECTRIS Ltd., Baden-Daettwil, Switzerland) was calibrated using standard diffraction patterns at a sample-to-detector distance of 1500 mm with readout noise of approximately 10 counts·s^−1^. The scattering vector range (*q*) was 0.14–3.80 nm^−1^ for all samples. Samples (approximately 1 mm thick) were loaded into sample holders and sealed with transparent tape. Scattering patterns were collected in single-frame mode with 5 min exposures and multiple readouts. Two-dimensional SAXS images were processed to one-dimensional data using *Fit2D* software (version is 1.0.0.4) [54], followed by further analysis using the *S* program package [55].

### 4.5. Adsorption Measurements

The single-component adsorption isotherms of CH_4_, CO_2_, and N_2_ were measured using a JWGB JW-BK300 adsorption analyser (Beijing Jingwei Gaobo Instrument Co., Ltd., Beijing, China). Before measurement, the samples were activated under vacuum at 120 °C for 3 h to remove adsorbed moisture and impurities. Gas adsorption tests were conducted at 273 and 298 K.

IAST calculations were performed using the single-component CO_2_ and CH_4_ adsorption isotherms measured at 298 K for an equimolar CO_2_/CH_4_ mixture. The CO_2_ isotherms were fitted using a dual-site Langmuir model, while the CH_4_ isotherms were fitted using either a Langmuir or Freundlich model according to the corresponding isotherm characteristics.

The multicomponent breakthrough performances were measured using a BTsorb-100 SMPPRO system from JWGB (Beijing Jingwei Gaobo Instrument Co., Ltd., Beijing, China). Before testing, the sample was activated at 300 °C for 6 h under a helium flow of 10 mL·min^−1^. Breakthrough tests were conducted at 298 K and 1 bar using a CO_2_/CH_4_ feed mixture of 50/50 (*v*/*v*). The helium carrier-gas flow rate was 4 mL min^−1^, the feed-gas flow rate was 2 mL min^−1^, and the argon makeup-gas flow rate was 10 mL min^−1^.

CO_2_ cyclic adsorption stability was evaluated using the BTsorb-100 SMPPRO system at 298 K and 1 bar. Before cycling, the sample was pretreated under helium at 300 °C for 6 h with a flow rate of 10 mL min^−1^. Desorption was performed by purging with helium at 298 K for 30 min at a flow rate of 10 mL min^−1^. The adsorption–desorption process was repeated for five cycles under the same conditions.

### 4.6. Simulation and Computational Methods

The initial CP structure was taken from the HEU framework topology in the Materials Studio database. The crystal information file of Mg-MOF-74 was obtained from the Cambridge Structural Database. The CP model was modified according to the experimentally determined framework and elemental composition. Specifically, six spatially separated Si sites within one unit cell were substituted by Al, and extra-framework Na^+^, K^+^, and Mg^2+^ cations were introduced to maintain framework charge balance and approximate the experimentally determined cation composition. The resulting CP unit-cell model was geometrically optimized using the CASTEP module in Materials Studio with the Perdew–Burke–Ernzerhof (PBE) functional within the generalized gradient approximation (GGA). The box dimensions of CP, Mg-MOF-74, and PSU@Mg-CP-3 h were 3.5802 × 3.14962 × 2.2162 nm, 3.5082 × 3.1490 × 2.2160 nm, and 3.5442 × 3.1493 × 5.1696 nm, respectively. Composite models were constructed as heterostructures to evaluate the influence of interfacial PSU growth on gas adsorption and separation. The Mg content of the composite models was constrained according to the experimentally determined AAS results. The constructed composite models were subsequently subjected to geometry optimization and molecular-dynamics relaxation before adsorption, diffusion, and interaction-energy calculations. These models were used as experimentally constrained structural representations for comparing the effects of interfacial PSU growth rather than as exact atom-by-atom reproductions of the experimental materials.

The subsequent classical-mechanics calculations were performed using the Forcite and Sorption modules in Materials Studio. The calculation quality was set to Fine, the COMPASS III force field was employed, and atomic charges were assigned using the corresponding force-field parameters. Single-component CO_2_, CH_4_, and N_2_ adsorption isotherms were calculated using fixed-pressure grand canonical Monte Carlo (GCMC) simulations in the Sorption module at 298 K over a pressure range of 1–100 kPa. The same force-field and charge-assignment settings were applied consistently to all structural models to enable direct comparison of their adsorption behavior.

Molecular-dynamics simulations were performed using the Forcite module. Each system was first simulated for 50 ps in the NVT ensemble using the Nosé thermostat with a time step of 1 fs, followed by a 1000 ps simulation in the NVE ensemble using the same time step. The NVE stage comprised 1,000,000 simulation steps, and one trajectory frame was saved every 10,000 steps for subsequent dynamic analysis. The relaxed configurations were used for the subsequent diffusion and interaction-energy calculations.

The self-diffusion coefficient (*D*) was calculated from the mean square displacement of the molecular center of mass according to the Einstein relation [56]:

(7) $$D=\frac{1}{2d}\underset{t\to \infty }{lim}\frac{\left({|r\left(t\right)-r\left(0\right)|}^{2}\right)}{t}$$

In Equation (7), *r(t)* is the center of mass position vector at time *t*, and *d* is the dimensionality of the system. The diffusion coefficient was obtained from the slope of the linear region of the mean square displacement curve. The kinetic selectivity was calculated according to Equation (8):

(8) $$S_{kinetic}=\frac{D_{A}}{D_{B}}$$

where *D_A_* and *D_B_* are the diffusion coefficients of components A and B, respectively.

The relaxed structural models were further used for interaction-energy calculations. DFT calculations were conducted using the Perdew–Burke–Ernzerhof (PBE) exchange-correlation functional within the generalized gradient approximation (GGA). The interaction energy (IE) values were calculated according to Equation (9):

(9) IE=EM+X−EM−EXn

where *E_M+X_* is the total energy of PSU@Mg-PCP with the adsorbed gas molecule (CO_2_, N_2,_ or CH_4_), *E_M_* is the energy of the PSU@Mg-PCP system without adsorbates, and *E_X_* is the energy of the gas molecule (CO_2_, N_2,_ or CH_4_), and *n* is the number of gas molecules.

## Figures and Tables

**Table 1:** Collections of the peak A energy positions at Mg K-edge, estimated Mg-O bond lengths, bond-valence parameters, calculated coordination number (CN) (Mg-O), and SAXS-derived *Ds* values.

Samples	Peak A Energy (eV)	*d_Mg-O_* (Å)	*ν_Mg-O_*	Calculated CN (Mg-O)	*D_s_* Value (Linear Fitting Range)
Mg-PCP	-	-	-	-	2.16 (−1.58~−1.44)
Mg-MOF-74	1310.4	2.01	0.42	4.76	2.82 (−1.62~−1.42)
PSU@Mg-PCP-10	1309.2	1.93	0.53	3.75	2.19 (−1.67~−1.54)
PSU@Mg-PCP-60	1309.5	1.94	0.51	3.94	2.23 (−1.62~−1.49)
PSU@Mg-PCP-180	1310.1	1.99	0.45	4.41	2.35 (−1.91~−1.77)

## Data Availability

Data will be made available on request.

## Supplementary Materials

The following supporting information can be downloaded at: https://www.mdpi.com/article/10.3390/gels12100902/s1, Scheme S1. Schematic illustration of PSU@Mg-PCP preparation. Figure S1. XRD patterns of CTAB-PCP (a), PCP (b), and NH_4_-PCP (c). Figure S2. Pore size distributions of Mg-PCP (a), Mg-MOF-74 (b), PSU@Mg-PCP-10 (c), PSU@Mg-PCP-60 (d), and PSU@Mg-PCP-180 (e), derived from the desorption data of N_2_ adsorption–desorption isotherms at 77 K. Figure S3. TG-DTG curves (A) of DHTA and the physical mixture of Mg-MOF-74 and Mg-PCP; DTG curves (B) of Mg-PCP (a), PSU@Mg-PCP-10 (b), PSU@Mg-PCP-60 (c), PSU@Mg-PCP-180 (d), and Mg-MOF-74 (e). Figure S4. SEM-EDS mappings of PSU@Mg-PCP-10 (A), PSU@Mg-PCP-60 (B), and PSU@Mg-PCP-180 (C). Figure S5. FT-IR spectra of Mg-PCP (a), PSU@Mg-PCP-10 (b), PSU@Mg-PCP-180 (c), and Mg-MOF-74 (d). Figure S6. Selectivity of CO_2_/CH_4_ (A,B), CO_2_/N_2_ (C,D) and CH_4_/N_2_ (E,F) at 273 K (A,C,E) and 298 K (B,D,F) for PSU@Mg-PCP-10 (a), PSU@Mg-PCP-60 (b), PSU@Mg-PCP-180 (c), Mg-PCP (d), and Mg-MOF-74 (e). Figure S7. Isosteric heats of adsorption (*Q_st_*) for CO_2_ (A), CH_4_ (B), and N_2_ (C): PSU@Mg-PCP-10 (a), PSU@Mg-PCP-60 (b), PSU@Mg-PCP-180 (c), Mg-PCP (d), and Mg-MOF-74 (e). Figure S8. Geometrically optimized structures of Mg-PCP (A), Mg-MOF-74 (B), disordered PSU@Mg-PCP (C), and ordered PSU@Mg-PCP (D). Figure S9. Simulated adsorption isotherms of CO_2_ (A), CH_4_ (B), and N_2_ (C) at 298 K on Mg-PCP (a), Mg-MOF-74 (b), disordered PSU@Mg-PCP (c), and ordered PSU@Mg-PCP (d). Figure S10. MSD plots of CO_2_/CH_4_ (A–D), CO_2_/N_2_ (E–H), and CH_4_/N_2_ (I–L) for Mg-PCP (A,E,I), Mg-MOF-74 (B,F,J), disordered PSU@Mg-PCP (C,G,K), and ordered PSU@Mg-PCP (D,H,L). Figure S11. Optimized conformational geometries and calculated IE values with CO_2_ (A), CH_4_ (B), and N_2_ (C). Table S1. Elemental contents and mass losses of Mg-PCP and PSU@Mg-PCP. composites. Table S2. Isosteric heats of adsorption (Qst) for CO_2_, CH_4_, and N_2_ on different samples. Table S3. Simulated kinetic selectivities of CO_2_/CH_4_, CO_2_/N_2_, and CH_4_/N_2_ for different structural models.
