# Supplementary material for: Confined Mg2+-Directed Interfacial Evolution of Primary Structural Units of Mg-MOF-74 on Platelet Clinoptilolite for CO2/CH4, CH4/N2, and CO2/N2 Separations
Source: Gels. 2026 Oct 7;12(10):902. doi: 10.3390/gels12100902 (PMC13704717; doi:10.3390/gels12100902)
Supplement: Supplementary file 1 [file gels-12-00902-s001.zip › gels-4570578-supplementary.pdf]

Confined Mg^{2+}-directed interfacial evolution of primary struc-tural units of Mg-MOF-74 on platelet clinoptilolite for CO_2/CH_4, CH_4/N_2, and CO_2/N_2 separations

Nan Sun, Tiejun Ma, Shengquan Liu, Tallat Munir, Bang Xu*, Jihong Sun*

Beijing Key Laboratory for Green Catalysis and Separation, Beijing University of Technology, Beijing, 100124, China

Electronic Supporting Information

Figure S1. XRD patterns of CTAB-PCP (a), PCP (b), and NH_4-PCP (c).

Figure S2. Pore size distributions of Mg-PCP (a), Mg-MOF-74 (b), PSU@Mg-PCP-10 (c), PSU@Mg-PCP-60 (d), and PSU@Mg-PCP-180 (e), derived from the desorption data of N_2 adsorption-desorption isotherms at 77 K.

Figure S3. TG-DTG curves (A) of DHTA and the physical mixture of Mg-MOF-74 and Mg-PCP; DTG curves (B) of Mg-PCP (a), PSU@Mg-PCP-10 (b), PSU@Mg-PCP-60 (c), PSU@Mg-PCP-180 (d), and Mg-MOF-74 (e).

Figure S4. SEM-EDS mappings of PSU@Mg-PCP-10 (A), PSU@Mg-PCP-60 (B), and PSU@Mg-PCP-180 (C).

Figure S5. FT-IR spectra of Mg-PCP (a), PSU@Mg-PCP-10 (b), PSU@Mg-PCP-180 (c), and Mg-MOF-74 (d).

Figure S6. Selectivity of CO_2/CH_4 (A, B), CO_2/N_2 (C, D) and CH_4/N_2 (E, F) at 273 K (A, C, E) and 298 K (B, D, F) for PSU@Mg-PCP-10 (a), PSU@Mg-PCP-60 (b),

PSU@Mg-PCP-180 (c), Mg-PCP (d), and Mg-MOF-74 (e).

Figure S7. Isosteric heats of adsorption (Q_{st}) for CO_2 (A), CH_4 (B), and N_2 (C): PSU@Mg-PCP-10 (a), PSU@Mg-PCP-60 (b), PSU@Mg-PCP-180 (c), Mg-PCP (d), and Mg-MOF-74 (e).

Figure S8. Geometrically optimized structures of Mg-PCP (A), Mg-MOF-74 (B), disordered PSU@Mg-PCP (C), and ordered PSU@Mg-PCP (D).

Figure S9. Simulated adsorption isotherms of CO_2 (A), CH_4 (B), and N_2 (C) at 298 K on Mg-PCP (a), Mg-MOF-74 (b), disordered PSU@Mg-PCP (c), and ordered PSU@Mg-PCP (d).

Figure S10. MSD plots of CO_2/CH_4 (A-D), CO_2/N_2 (E-H), and CH_4/N_2 (I-L) for Mg-PCP (A, E, I), Mg-MOF-74 (B, F, J), disordered PSU@Mg-PCP (C, G, K), and ordered PSU@Mg-PCP (D, H, L).

Figure S11. Optimized conformational geometries and calculated IE values with CO_2 (A), CH_4 (B), and N_2 (C).

Scheme S1. Schematic illustration of PSU@Mg-PCP preparation.

Table S1. Elemental contents and mass losses of Mg-PCP and PSU@Mg-PCP. composites.

Table S3. Simulated kinetic selectivities of CO_2/CH_4, CO_2/N_2, and CH_4/N_2 for different structural models.

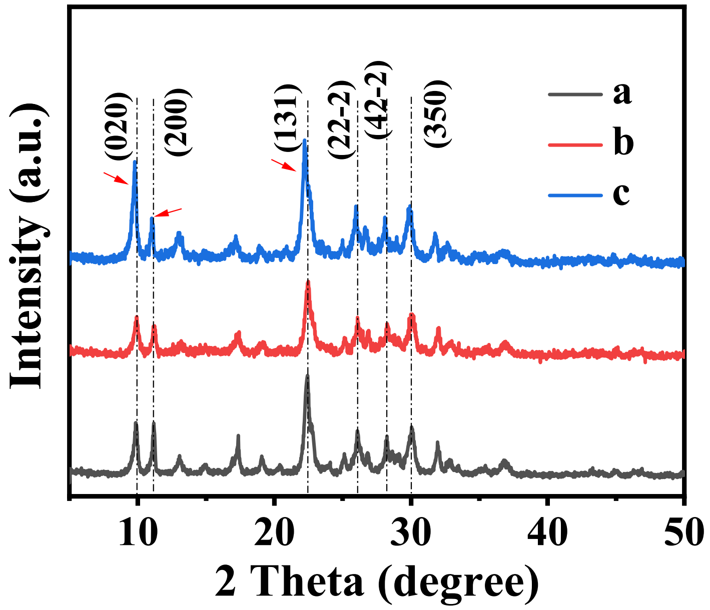

Figure S1. XRD patterns of CTAB-PCP (a), PCP (b), and NH_4-PCP (c).

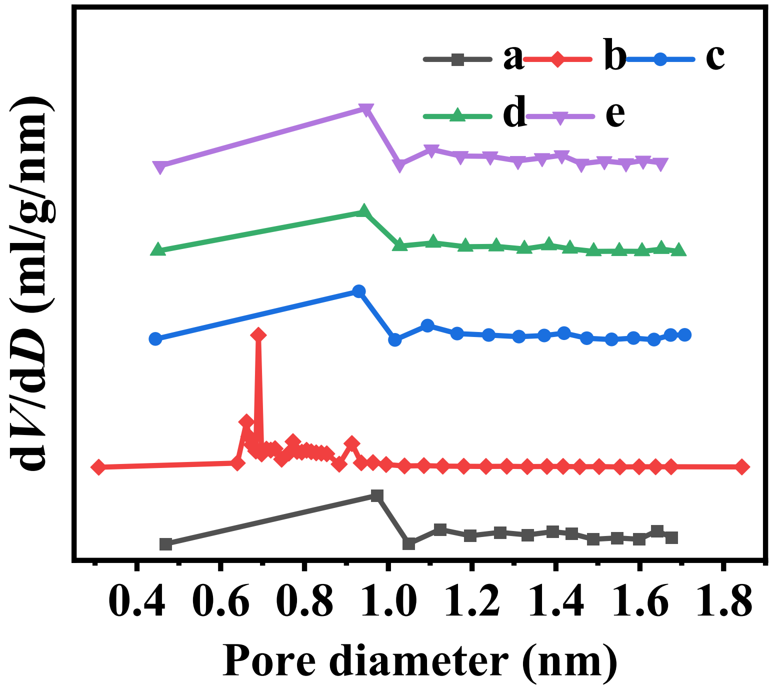

Figure S2. Pore size distributions of Mg-PCP (a), Mg-MOF-74 (b), PSU@Mg-PCP-10 (c), PSU@Mg-PCP-60 (d), and PSU@Mg-PCP-180 (e), derived from the desorption data of N_2 adsorption-desorption isotherms at 77 K.

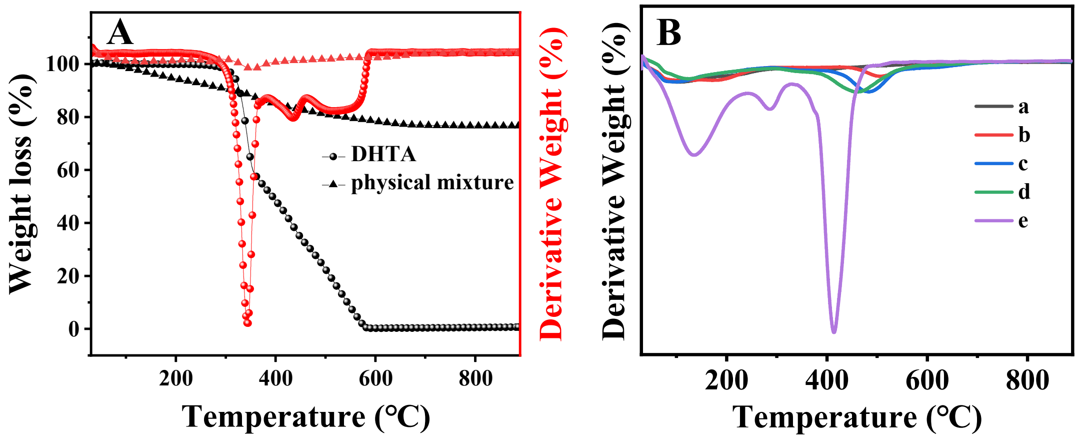

Figure S3. TG-DTG curves (A) of DHTA and the physical mixture of Mg-MOF-74 and Mg-PCP; DTG curves (B) of Mg-PCP (a), PSU@Mg-PCP-10 (b), PSU@Mg-PCP-60 (c), PSU@Mg-PCP-180 (d), and Mg-MOF-74 (e).

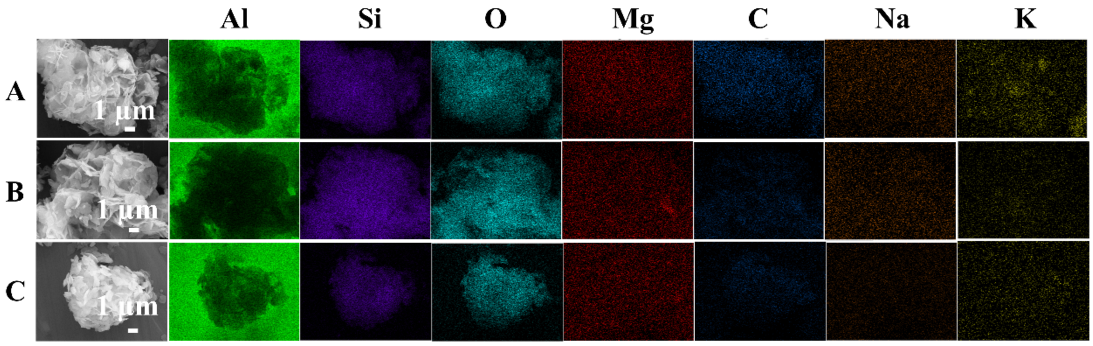

Figure S4. SEM-EDS mappings of PSU@Mg-PCP-10 (A), PSU@Mg-PCP-60 (B), and PSU@Mg-PCP-180 (C).

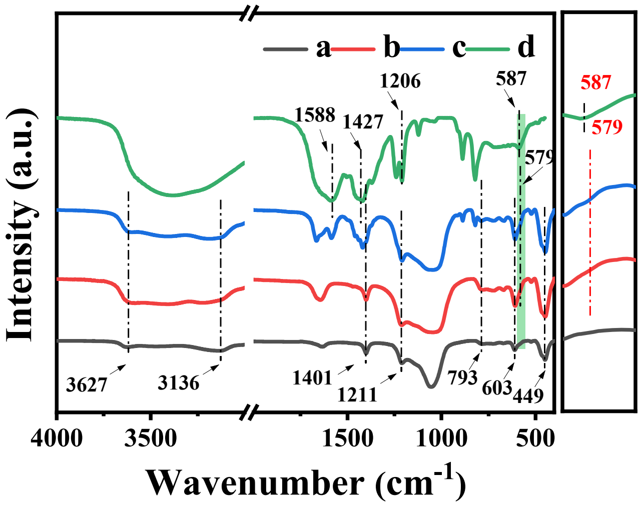

Figure S5. FT-IR spectra of Mg-PCP (a), PSU@Mg-PCP-10 (b), PSU@Mg-PCP-180 (c), and Mg-MOF-74 (d).

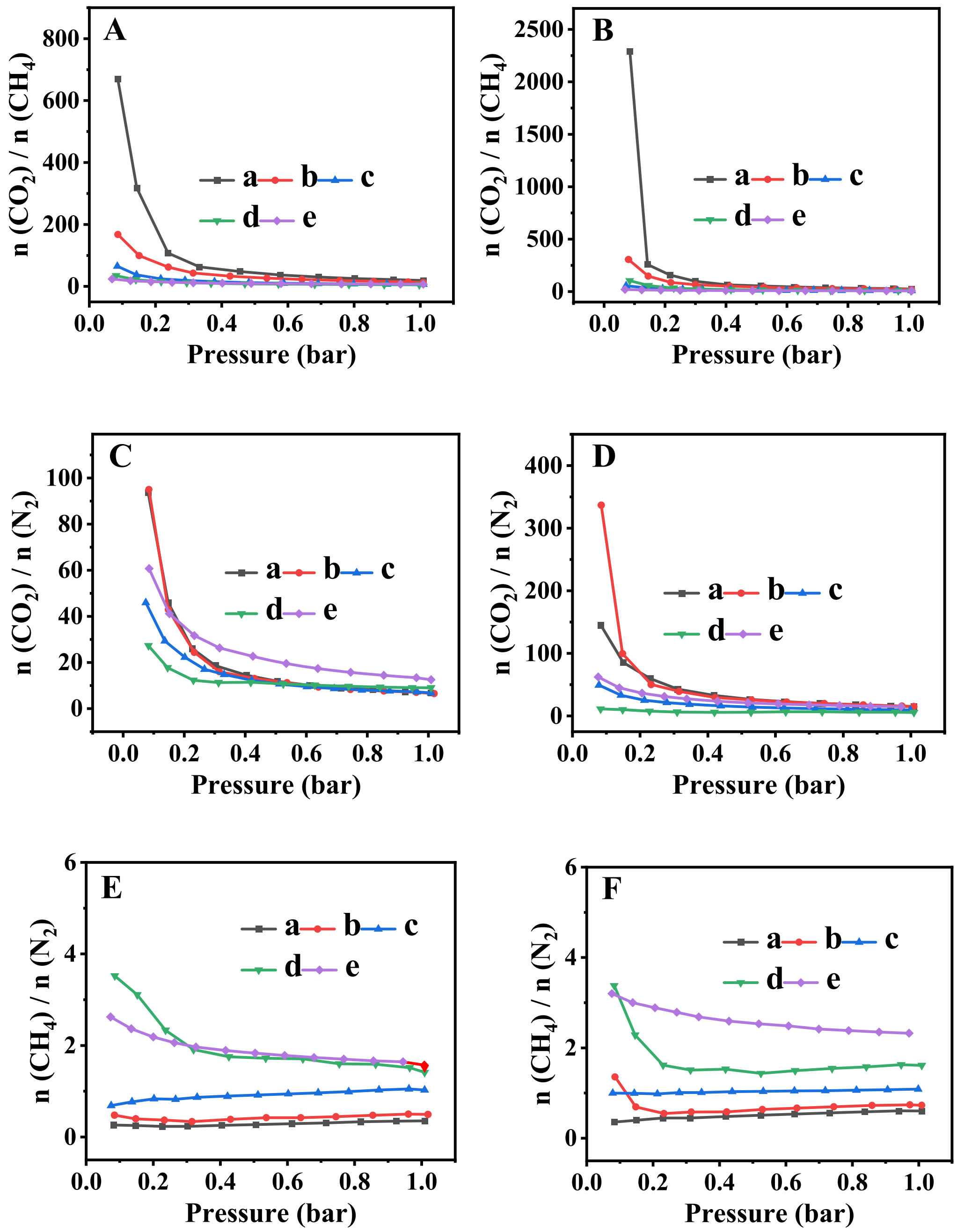

Figure S6. Selectivity of CO_2/CH_4 (A, B), CO_2/N_2 (C, D) and CH_4/N_2 (E, F) at 273 K (A, C, E) and 298 K (B, D, F) for PSU@Mg-PCP-10 (a), PSU@Mg-PCP-60 (b), PSU@Mg-PCP-180 (c), Mg-PCP (d), and Mg-MOF-74 (e).

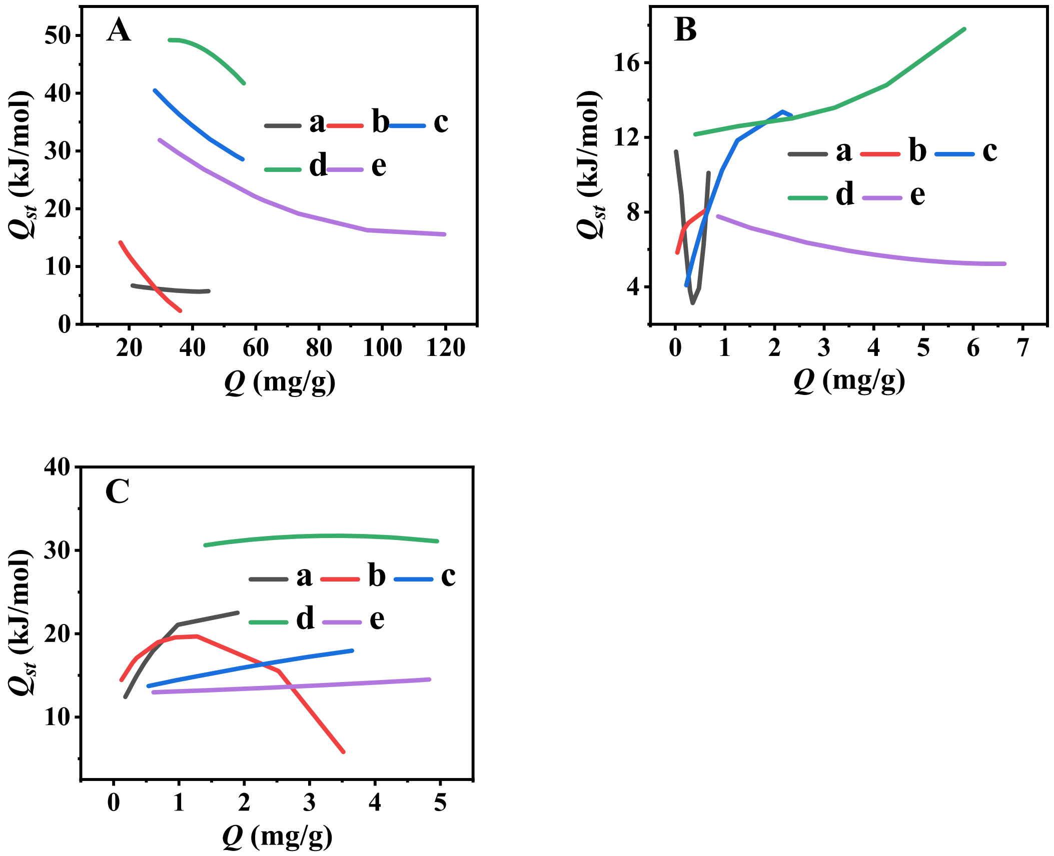

Figure S7. Isosteric heats of adsorption (Q_{st}) for CO_2 (A), CH_4 (B), and N_2 (C): PSU@Mg-PCP-10 (a), PSU@Mg-PCP-60 (b), PSU@Mg-PCP-180 (c), Mg-PCP (d), and Mg-MOF-74 (e).

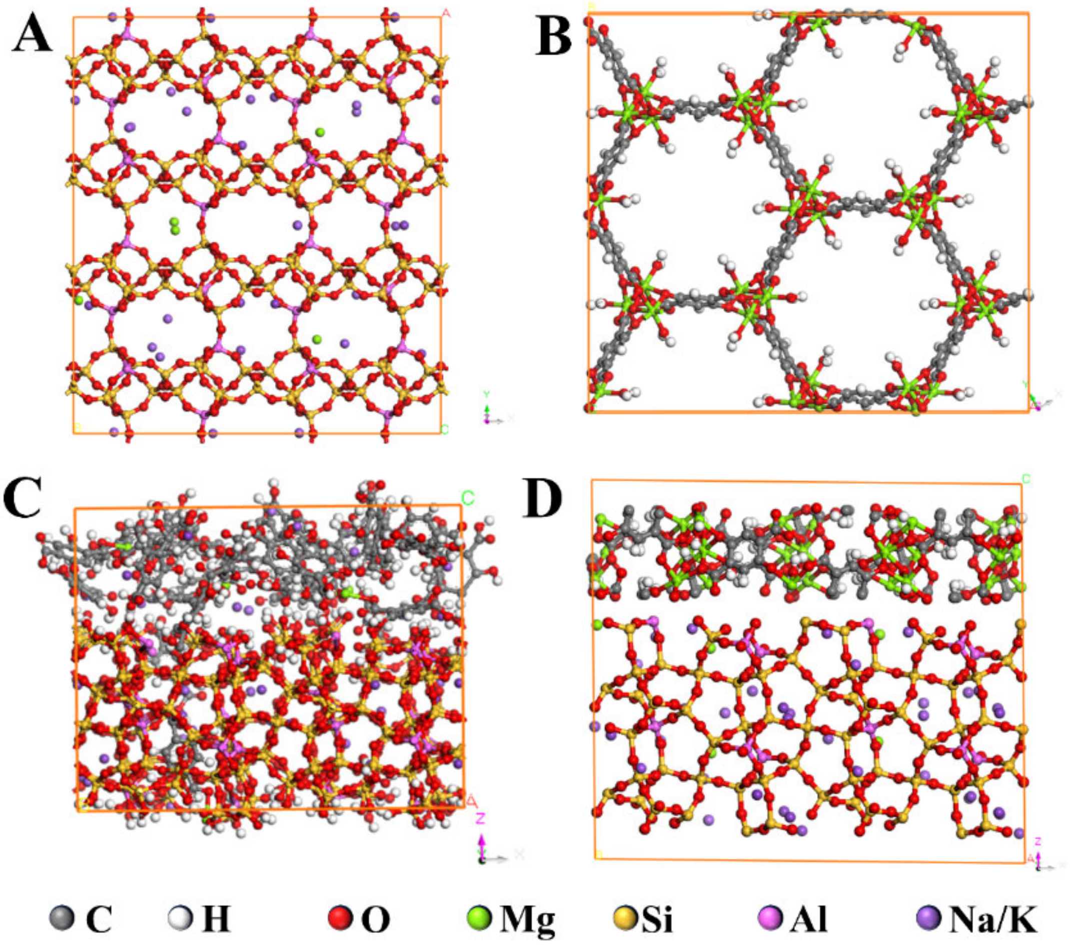

Figure S8. Geometrically optimized structures of Mg-PCP (A), Mg-MOF-74 (B), disordered PSU@Mg-PCP (C), and ordered PSU@Mg-PCP (D).

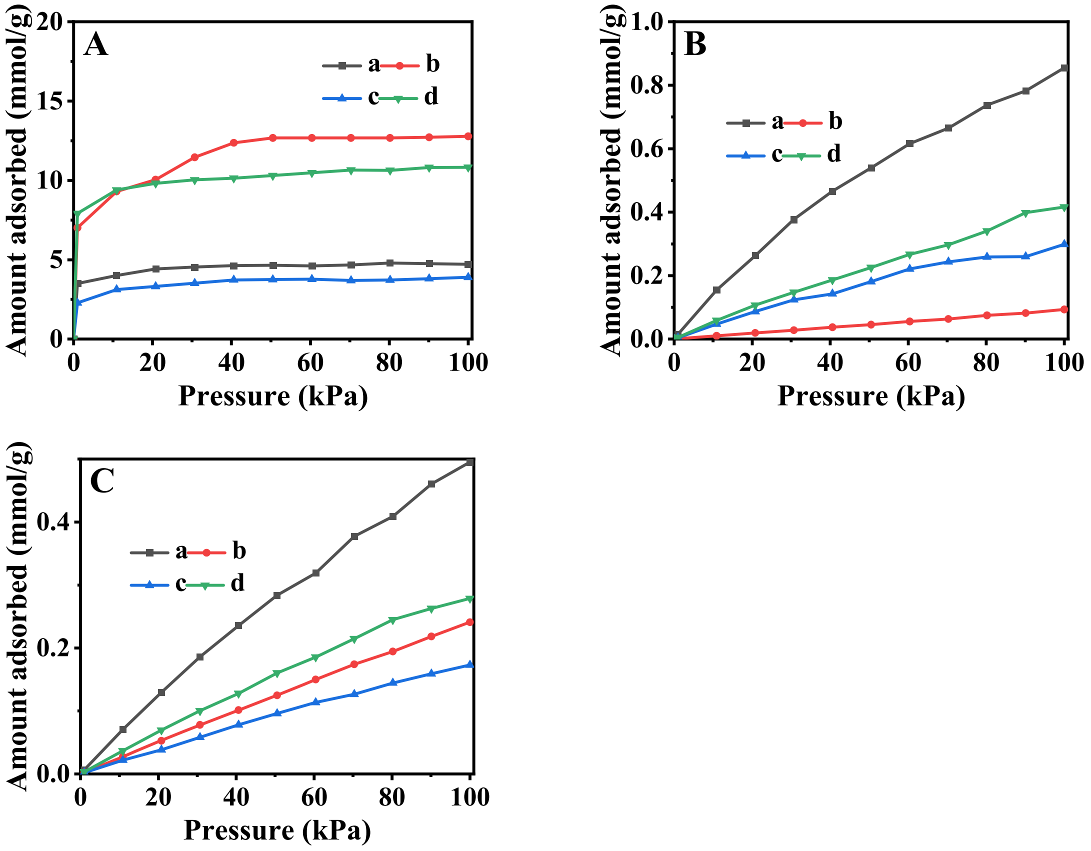

Figure S9. Simulated adsorption isotherms of CO_2 (A), CH_4 (B), and N_2 (C) at 298 K on Mg-PCP (a), Mg-MOF-74 (b), disordered PSU@Mg-PCP (c), and ordered PSU@Mg-PCP (d).

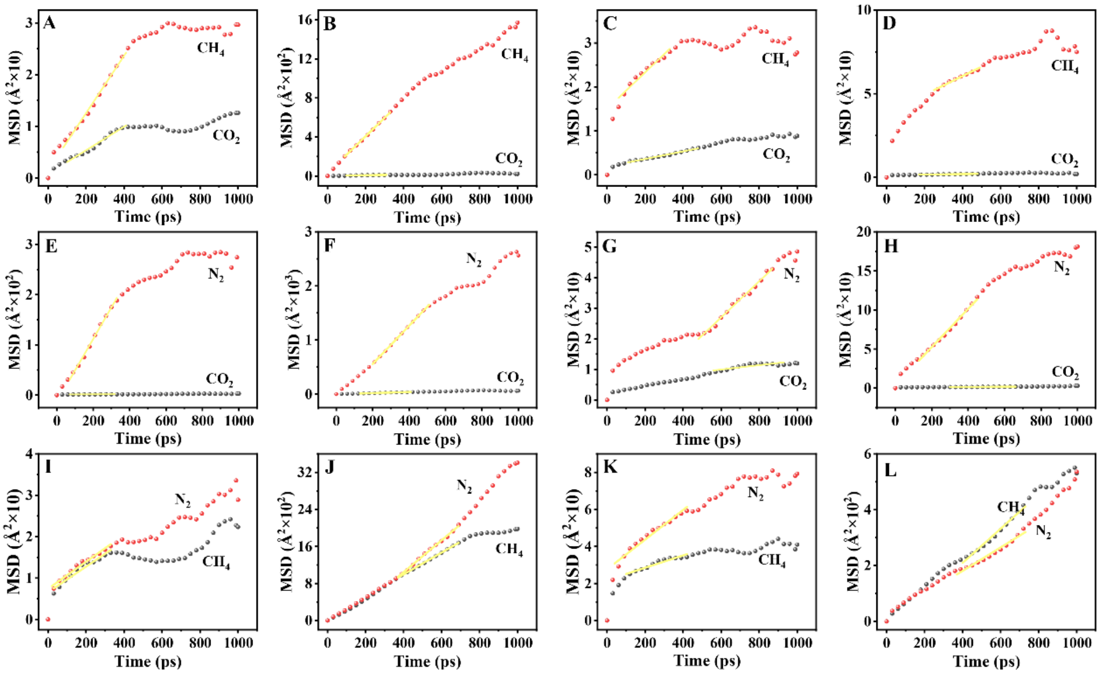

Figure S10. MSD plots of CO_2/CH_4 (A-D), CO_2/N_2 (E-H), and CH_4/N_2 (I-L) for Mg-PCP (A, E, I), Mg-MOF-74 (B, F, J), disordered PSU@Mg-PCP (C, G, K), and ordered PSU@Mg-PCP (D, H, L).

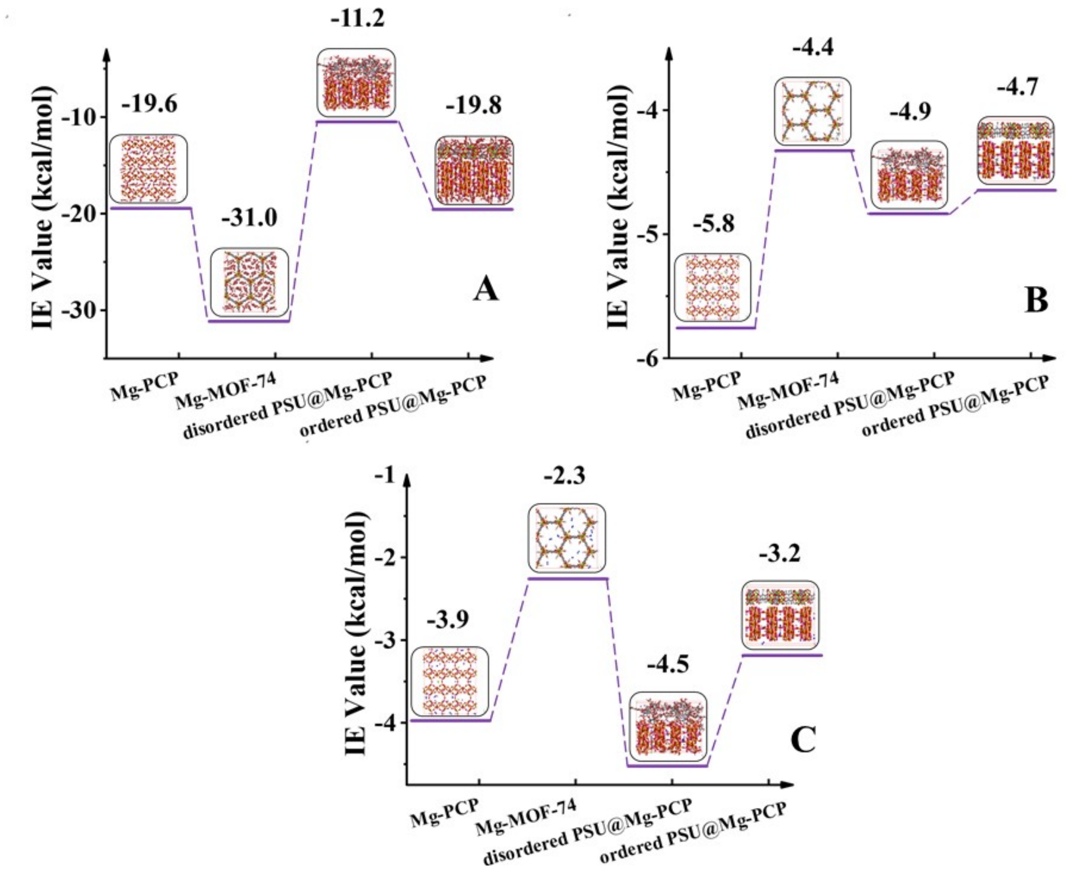

Figure S11. Optimized conformational geometries and calculated *IE* values with CO_2 (A), CH_4 (B), and N_2 (C).

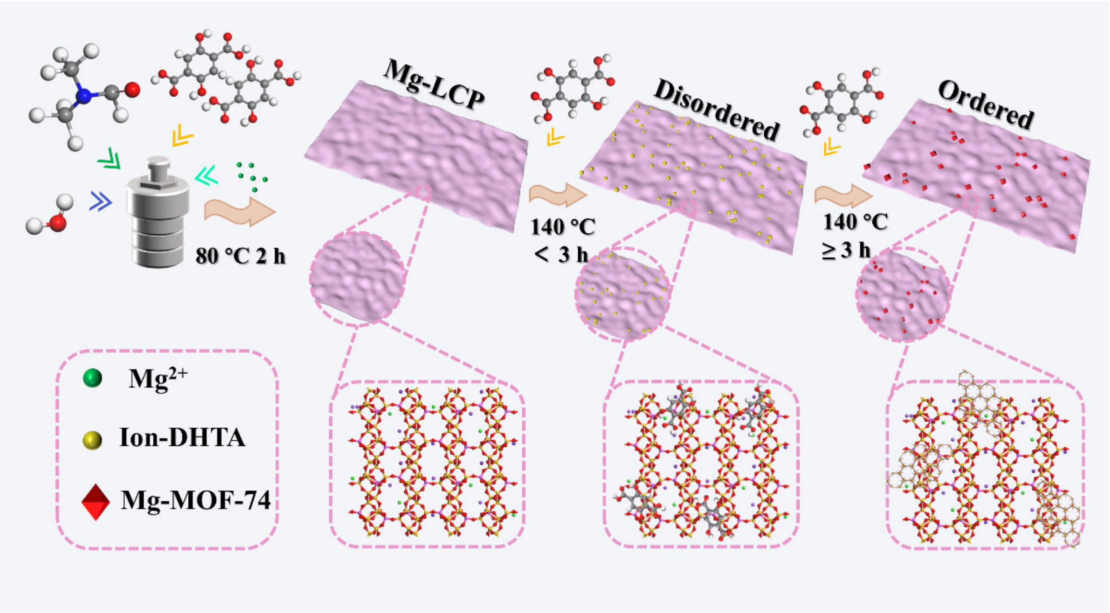

Scheme S1. Schematic illustration of PSU@Mg-PCP preparation.

Table S1. Elemental contents and mass losses of Mg-PCP and PSU@Mg-PCP. composites.

Samples	Si[a]	Al[a]	O[a]	Mg[a]	C[a]	Na[a]	K[a]	Mg[b]	Mg[c]	Total loss, 30-900 °C	Loss <300 °C	Loss >300 °C
Mg-PCP	-	-	-	-	-	-	-	1.10	-	19.2	-	-
PSU@Mg-PCP-10	13.97	47.8	21.45	0.56	15.56	0	0.65	1.06	1.03	17.47	10.17	7.30
PSU@Mg-PCP-60	19.5	33.47	28.73	0.57	17.42	0.03	0.32	1.05	-	21.67	8.91	12.76
PSU@Mg-PCP-180	6.67	67.82	9.99	0.15	15.29	0	0	1.11	0.93	20.58	7.15	13.37

All elemental compositions and mass losses are given in wt%.

[a] Determined by SEM-EDS. [b] Determined by AAS. [c] Determined by XPS.

Table S2. Isosteric heats of adsorption (*Qst*) for CO_2, CH_4, and N_2 on different samples.

Samples	Q_s/(kcal·mol^{-1})	Q_s/(kcal·mol^{-1})	Q_s/(kcal·mol^{-1})
	CO_2	CH_4	N_2
Mg-PCP	41.71	17.79	31.08
Mg-MOF-74	31.88	7.77	14.50
PSU@Mg-PCP-10	6.70	11.24	22.51
PSU@Mg-PCP-60	14.15	8.15	19.67
PSU@Mg-PCP-180	40.48	13.37	17.97

Table S3. Simulated kinetic selectivities of CO_2/CH_4, CO_2/N_2, and CH_4/N_2 for different structural models.

Samples	kinetic selectivitives		
	CO_2 /CH_4	CO_2 /N_2	CH_4 /N_2
Mg-PCP	0.4118	0.0027	0.9488
Mg-MOF-74	0.0179	0.0292	0.6842
disordered PSU@MgPCP	0.2084	0.0997	0.0417
ordered PSU@MgPCP	0.0294	0.00514	1.4841
